# Age‐associated de‐repression of retrotransposons in the *Drosophila* fat body, its potential cause and consequence

**DOI:** 10.1111/acel.12465

**Published:** 2016-04-12

**Authors:** Haiyang Chen, Xiaobin Zheng, Danqing Xiao, Yixian Zheng

**Affiliations:** ^1^ Key Laboratory of Gene Engineering of the Ministry of EducationState Key Laboratory of BiocontrolSchool of Life SciencesSun Yat‐sen UniversityGuangzhou510275China; ^2^ Department of EmbryologyCarnegie Institution for ScienceBaltimoreMD21218USA

**Keywords:** aging, DNA damage, heterochromatin, Lamin‐B, lamin Dm0, retrotransposons

## Abstract

Eukaryotic genomes contain transposable elements (TE) that can move into new locations upon activation. Since uncontrolled transposition of TEs, including the retrotransposons and DNA transposons, can lead to DNA breaks and genomic instability, multiple mechanisms, including heterochromatin‐mediated repression, have evolved to repress TE activation. Studies in model organisms have shown that TEs become activated upon aging as a result of age‐associated deregulation of heterochromatin. Considering that different organisms or cell types may undergo distinct heterochromatin changes upon aging, it is important to identify pathways that lead to TE activation in specific tissues and cell types. Through deep sequencing of isolated RNAs, we report an increased expression of many retrotransposons in the old *Drosophila* fat body, an organ equivalent to the mammalian liver and adipose tissue. This de‐repression correlates with an increased number of DNA damage foci and decreased level of *Drosophila* lamin‐B in the old fat body cells. Depletion of the *Drosophila* lamin‐B in the young or larval fat body results in a reduction of heterochromatin and a corresponding increase in retrotransposon expression and DNA damage. Further manipulations of lamin‐B and retrotransposon expression suggest a role of the nuclear lamina in maintaining the genome integrity of the *Drosophila* fat body by repressing retrotransposons.

## Introduction

Epigenetic modifications of chromatin are critical for many biological functions ranging from organism development to tissue homeostasis. Studies in multiple organisms have shown that maintaining the repressive epigenetic state of heterochromatin is important for lifespan extension. For example, pioneering studies in budding yeast have shown that replicative aging is correlated with heterochromatin loss in telomeric regions, mating type loci, and rDNA repeats (Kim *et al*., 1996; Smeal *et al*., 1996; Kennedy *et al*., 1997). Importantly, overexpression of Sir2, a histone deacetylase required for heterochromatin formation and gene silencing, induces an increased lifespan in yeast (Kaeberlein *et al*., 1999). The connection between heterochromatin maintenance and lifespan extension is further strengthened by studies in *Caenorhabditis elegans* and *Drosophila* (Rogina & Helfand, 2004; Hashimoto *et al*., 2010; Jiang *et al*., 2013; Whitaker *et al*., 2013). Disruption of UTX‐1, a histone demethylase that reduces the heterochromatin epigenetic modification called histone H3 K27 trimethylation (H3K27me3), in *C. elegans* leads to increased H3K27me3 levels and prolonged lifespan (Jin *et al*., 2011; Maures *et al*., 2011). Moreover by modulating the expression of HP1, a protein required for the maintenance of heterochromatin, researchers have shown that increase or decrease HP1 levels leads to lifespan extension or shortening, respectively, in *Drosophila* (Larson *et al*., 2012). Consistently, old *Drosophila* exhibits both HP1 reduction and the reduction of pericentric heterochromatin as judged by a decrease in H3K9me3 modification (Wood *et al*., 2010; Larson *et al*., 2012; Chen *et al*., 2014).

Despite a clear connection between heterochromatin maintenance and lifespan extension (Feser *et al*., 2010; Meister *et al*., 2011; Larson *et al*., 2012; Jiang *et al*., 2013), the cause of heterochromatin loss and how it leads to cellular damage and aging is not well understood. Generally speaking, heterochromatin loss would lead to a mis‐expression of genes that are normally repressed, which could result in age‐ associated cellular defects. Heterochromatin is also enriched for transposable elements (TE). As mobile elements, TEs, including both the retrotransposons and DNA transposons, can transpose from one chromatin location to another (Wicker *et al*., 2007). In doing so, they create DNA breaks that can lead to genome damages. Since accumulation of DNA damage is known to cause cellular senescence and organismal aging (Best, 2009), activation of TEs could contribute to organismal aging. Although TE activation during development has also been implicated as important for proper development of the nervous system (Reilly *et al*., 2013), in most settings TEs are repressed by the heterochromatin state to prevent uncontrolled transposition (Levin & Moran, 2011; Wood & Helfand, 2013). Therefore, in addition to de‐repression of genes, the age‐associated heterochromatin loss could also cause an increased expression of TEs, which would in turn contribute to age‐associated cell and organ defects.

Consistent with the idea that TE activation could contribute toward aging (De Cecco *et al*., 2013b; Sedivy *et al*., 2013), increased expression of *Ty1* retrotransposon is correlated with increased chromosome rearrangements and genome instability in old yeasts (Maxwell *et al*., 2011; VanHoute & Maxwell, 2014; Patterson *et al*., 2015).

Additionally, increased expression of *Cer1*, which belongs to the *gypsy/Ty3* family of retrotransposons, is observed in old *C. elegans* (Dennis *et al*., 2012). Studies of the old *Drosophila* brains have also revealed an increased expression of several TEs, including the *gypsy* retrotransposon (Li *et al*., 2013). More recent studies have reported increased expression of *Alu* retrotransposons in the retinal pigmented epithelial cells from old humans, which can contribute to age‐associated macular degeneration (Kaneko *et al*., 2011; Tarallo *et al*., 2012). Interestingly, increased expression of *Alu* retrotransposons also occur when human adult stem cells undergo senescence (Wang *et al*., 2011; De Cecco *et al*., 2013a), which correlates with increased formation of DNA damage foci within the TE‐enriched chromatin regions.

Considering the connection between TE activation and aging, it is important to understand what cellular changes during aging may contribute to heterochromatin loss and TE activation. Since mutations in some heterochromatin modifying enzymes have been shown to regulate the lifespan of *C. elegans* and *Drosophila* (Siebold *et al*., 2010; Jin *et al*., 2011; Maures *et al*., 2011), it is possible that age‐associated changes of chromatin regulators could contribute to heterochromatin loss and TE activation. Nuclear lamins, the major structural components of the nuclear lamina that associate with the nuclear peripheral heterochromatin, are believed to contribute to the establishment and maintenance of the heterochromatin (Dechat *et al*., 2008; Bank & Gruenbaum, 2011; Kim *et al*., 2011; Meuleman *et al*., 2013). For example, lamins can help maintain heterochromatin by interacting with other heterochromatin regulators such as HP1 (Dechat *et al*., 2008; Bank & Gruenbaum, 2011). Interestingly, of the two A‐ and B‐types of lamins, lamin‐B1 (B‐type) has been shown to undergo age‐ associated reduction in human skin keratinocytes (Dreesen *et al*., 2013). A similar reduction of the single *Drosophila* lamin‐B protein, LAM (also called lamin Dm0), is observed in the old *Drosophila* fat body, a humoral immune organ that is equivalent to the liver and adipose tissue in mammals (Chen *et al*., 2014). The age‐associated LAM reduction in fat body leads to gut hyperplasia in *Drosophila*. This suggests that LAM loss upon aging could contribute to disease. Although LAM reduction in fat body is shown to lead to the de‐repression of immune responsive genes, it remains unclear whether such loss also influences TE repression.

Using genome‐wide transcriptome analyses (RNA sequencing, RNA‐seq), we have obtained the expression profiles of retrotransposons in young and old *Drosophila* fat bodies. Our analyses show that the old fat bodies exhibit a significant up‐regulation of a large number of retrotransposons. Our further studies suggest that LAM loss upon aging could contribute to increased retrotransposon expression and DNA damage due, in part, to the loss of heterochromatin. We will discuss our finding in the context of age‐associated pathologies.

## Results

### Increased expression of retrotransposons in old *Drosophila* fat bodies

**Figure 1:**
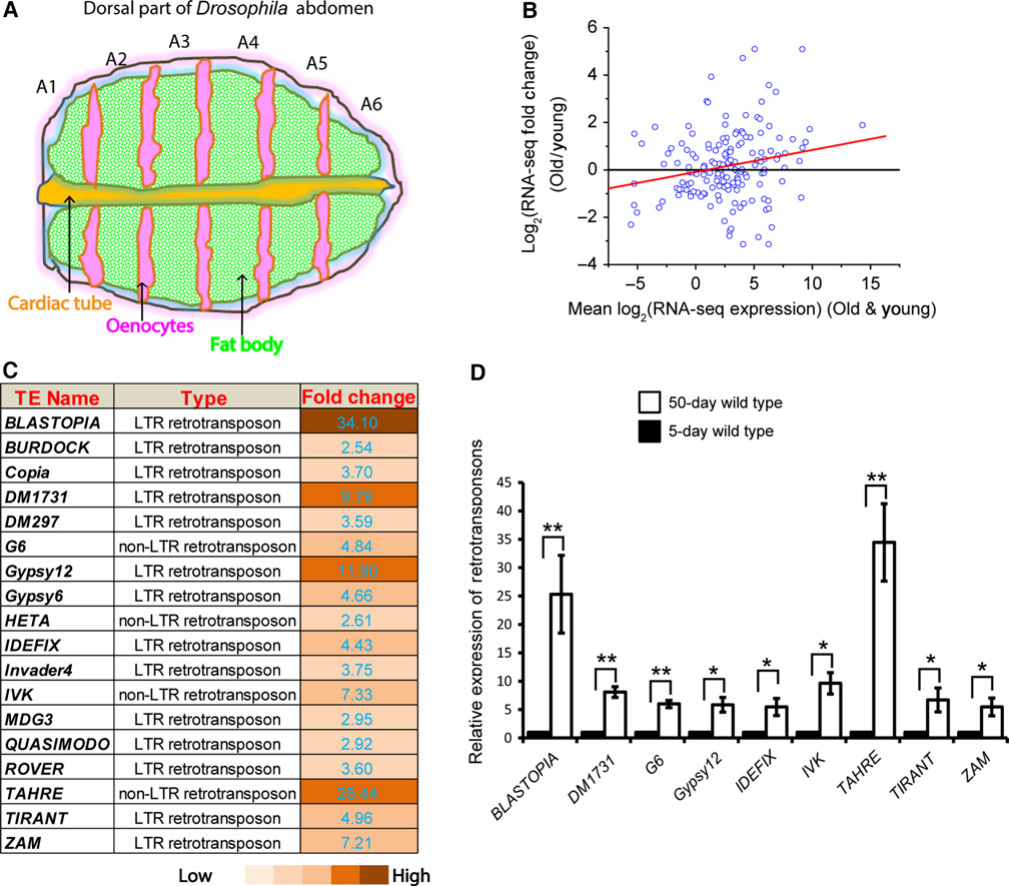
Age‐associated up‐regulation of retrotransposon expression in *Drosophila* fat bodies. (A) A cartoon illustration of adult *Drosophila* dorsal abdomen. Adult fat body cells (green) align the inner cuticle surface of abdominal segments (A1‐6). The oenocytes (pink) and cardiac tube (orange) are also shown. All immunofluorescence images of the fat body cells in this manuscript were captured from the A4 region. (B) MA‐plot comparing the retrotransposon RNA‐seq data between young and old fly fat bodies. Each dot represents one retrotransposon. *X*‐axis shows the mean RNA‐seq expression level of retrotransposons in young and old fat bodies, calculated by log2 (reads per million reads). *Y*‐axis shows the mean log2 expression change of retrotransposons in old fat bodies compared to young. The red line is the linear regression, showing that retrotransposons expressed at high levels in young and/or old fat bodies are more likely to be further up‐regulated in old fat bodies. (C) A list of retrotransposons that are up‐regulated by more than 2‐folds in old fat bodies compared to young. The numbers and color codes indicate the fold changes. (D) qRT‐PCR analyses of the indicated retrotransposons that was found to be up‐regulated by RNA‐seq in old fat bodies. The fold expression change of 50‐day fat bodies was plotted relative to 5‐day fat bodies, which was set to 1. Error bars, standard error of the mean (SEM) based on three independent experiments. Student's t‐tests: *P < 0.05, **P < 0.01.

Retrotransposons represent a major population of TEs in mammals and their de‐regulation leads to many of the reported TE‐associated human diseases (Hancks & Kazazian, 2012). Since retrotransposon activation is associated with their increased transcription, we performed RNA‐seq using dissected *Drosophila* fat bodies (Fig. 1A) from young (5 days) and old (50 days) animals. By analyzing the differential expression of 111 annotated *Drosophila* retrotransposons, we found a 2.7‐fold higher level of overall expression of total retrotransposons in old fat bodies than that of the young (Fig. 1B). Further analyses showed that 18 retrotransposons were significantly up‐regulated in old fat bodies (fold change>2, FDR < 0.05, Fig. 1C). These include 14 long terminal repeat (LTR) retrotransposons and four non‐LTR retrotransposons. We also found 18 down‐regulated retrotransposons upon aging, but both their overall expression level and the degree of down‐regulation are low (Fig. 1B, Table S1). By contrast, the fold increase is high in the 18 up‐regulated retrotransposons (Fig. 1B,C). Quantitative reverse transcription polymerase chain reaction (qRT‐PCR) analyses further confirmed the age‐associated de‐repression of retrotransposons in fat bodies (Fig. 1D). Thus upon aging, ~16% of the annotated retrotransposons exhibit significant up‐regulation in the *Drosophila* fat bodies.

### Increased retrotransposon expression correlates with elevated DNA damage signature in old *Drosophila* fat bodies

**Figure 2:**
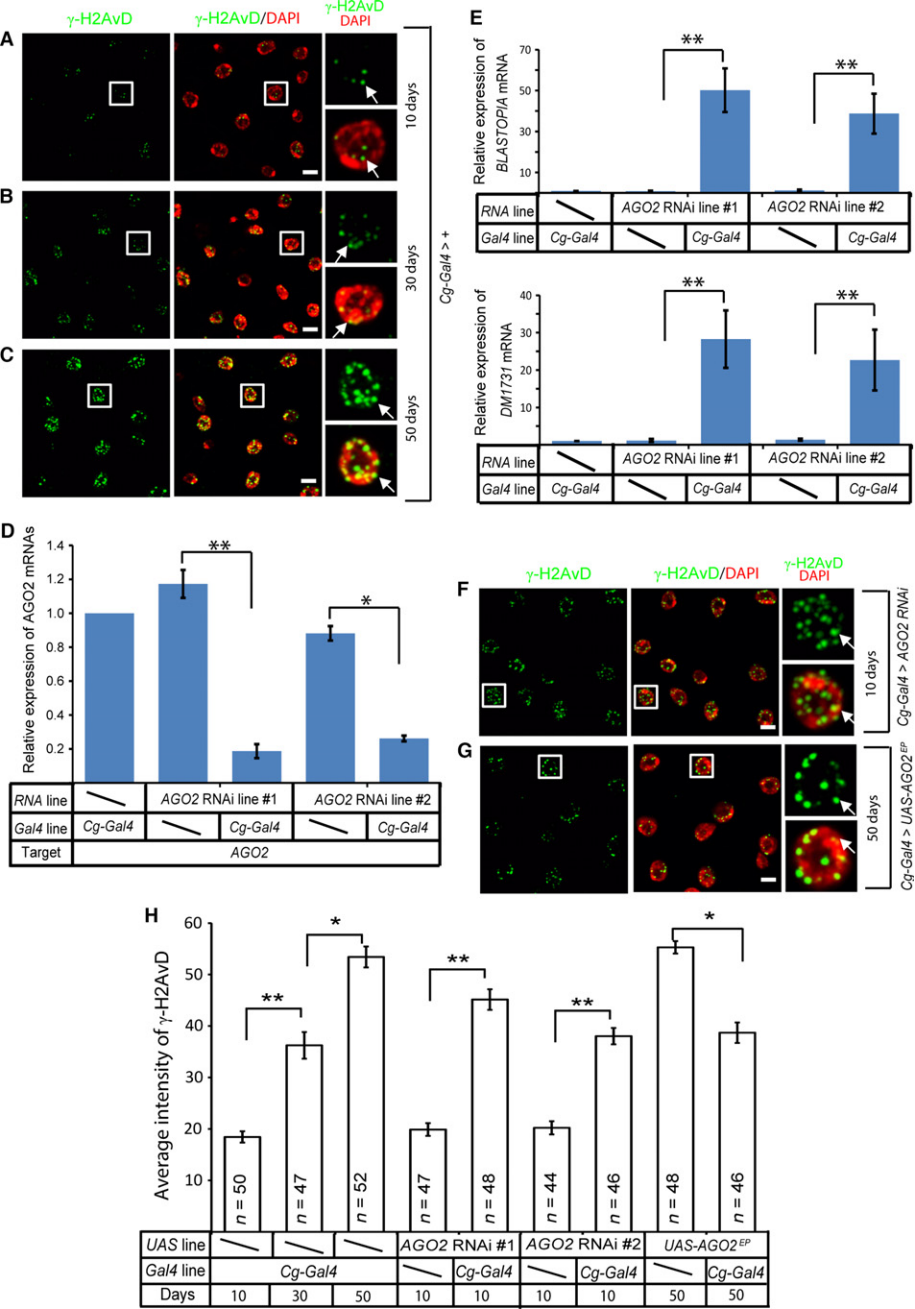
Retrotransposon de‐repression leads to DNA damage in old fat bodies. (A–C) Representative immunostaining images of γ‐H2AvD (green) and DNA (DAPI, red) labeling in fat bodies (from the A4 region). 10‐day‐old young control fat body cells (A, *Cg‐gal4/+;tub‐gal80* ^*ts*^ */+*) had a few faint nuclear γ‐H2AvD signals, which was gradually increased by 30 (B) and 50 days (C). Nuclei outlined by white squares are enlarged to the right. White arrows indicate γ‐H2AvD staining foci. Scale bars, 20 μm. (D) Two independent fly lines carrying fat body specific RNAi (Bloomington Stock Center, #34799 and #55672) driven by *Cg‐gal4;tub‐gal80* ^*ts*^ exhibited significant reduction of *AGO2* in fat body. The fold change of *AGO2* expression was determined relative to the Gal4 control flies (*Cg‐gal4/+;tub‐gal80* ^*ts*^ */+*), which was set to 1. Error bars, SEM based on three independent experiments. Student's t‐tests: *P < 0.05, **P < 0.01. (E) Depletion of *AGO2* in the fat body by the two different *AGO2* RNAi allele (*Cg‐ gal4/AGO2 RNAi#1*;*tub‐gal80* ^*ts*^ */+ and Cg‐gal4/AGO2 RNAi#2*;*tub‐gal80* ^*ts*^ */+*) significantly increased the expression of two retrotransposons (*BLASTOPIA* and *DM1731*) that were found to undergo age‐associated up‐regulation in fat body. The fold expression change was determined relative to the Gal4 control flies (*Cg‐ gal4/+;tub‐gal80* ^*ts*^ */+*), which was set to 1. Error bars, SEM based on three independent experiments. Student's t‐tests: *P < 0.05, **P < 0.01. (F–G) Depletion of *AGO2* (*Cg‐gal4/AGO2 RNAi#1*;*tub‐gal80* ^*ts*^ */+*) by RNAi in fat bodies increased γ‐H2AvD signal in 10‐day‐old young fat bodies (F), whereas the expression of *AGO2* (*Cg‐gal4/+;tub‐gal80ts/ UAS‐AGO2* ^*EP*^) in fat bodies reduced γ‐H2AvD signal in 50‐day old young fat bodies (G). Nuclei outlined by white squares are enlarged to the right. White arrows indicate γ‐H2AvD staining foci. Scale bars, 20 μm. (H) Quantification for γ‐H2AvD intensities in fat body cells from 10‐, 30‐, or 50‐day old Gal4 control flies (*Cg‐gal4/+;tub‐gal80* ^*ts*^ */+*), UAS control flies (for *AGO2* RNAi#1, *AGO2* RNAi#2, and *AGO2* EP line), 10‐day‐old flies with *AGO2* depleted in fat body (*Cg‐gal4/AGO2 RNAi#1;tub‐gal80* ^*ts*^ */+ and Cg‐gal4/AGO2 RNAi#2;tub‐ gal80* ^*ts*^ */+*), and 50‐day old flies with extra *AGO2* expression in fat body (*Cg‐ gal4/+;tub‐gal80* ^*ts*^ */UAS‐AGO2* ^*EP*^). The γ‐H2AvD signal was determined by measuring the total nuclear immunofluorescence signal from the images captured using the same microscopy settings (see the detailed description in the experimental procedures). Nuclei from the A4 region (see Fig. 1A) were measured to allow the comparison among different fat bodies. Error bars, SEM. Student's t‐test: *P < 0.05, **P < 0.01. The numbers (*n*) of fat body cells analyzed shown were from three independent experiments.

Retrotransposon expression can lead to their replication and insertion back into the genome, which would lead to increased DNA breaks. Therefore, we compared DNA damage foci between the old and young fat bodies. Using an antibody to *Drosophila* γ‐H2AvD (homologous to mammal γ‐H2AX) known to label double stranded DNA breaks, we visualized and quantified the total intensity of the nuclear DNA damage foci. These analyses revealed a gradual increase of nuclear γ‐H2AvD foci in 10, 30, and 50‐day old fat bodies (Fig. 2A–C,H). Therefore, the increased expression of retrotransposons upon aging is correlated with an increased accumulation of γ‐H2AvD foci in the nuclei of old *Drosophila* fat body cells.

We then asked whether forced activation of retrotransposons in young fat bodies could also lead to increased DNA damage as marked by γ‐H2AvD. The endogenous siRNA pathway mediated by *Dicer‐2* (*Dcr‐2*) and *Argonaute2* (*AGO2*) have been shown to repress retrotransposon expression in *Drosophila* somatic cells. Previous studies have shown that *AGO2* mutant flies or shRNA‐mediated depletion of *AGO2* caused increased expression of retrotransposons in *Drosophila* brain and S2 cells (Czech *et al*., 2008; Ghildiyal *et al*., 2008; Li *et al*., 2013). Thus, we depleted *AGO2* in the fat body (*Cg‐gal4>AGO2* shRNA) by shRNA (mediated by *Dicer‐1*‐ *AGO1*) using two different fly RNAi lines (line 1: Bloomington Stock Center #34799, and line 2: Bloomington Stock Center #55672) that target different *AGO2* sequences. We found that both lines resulted in a similar *AGO2* depletion (Fig. 2D). Using qRT‐ PCR, we found that depleting *AGO2* in 10‐day‐old fat bodies caused a significant increase in the expression of two retrotransposons (Fig. 2E) that we found to undergo age‐associated up‐regulation (see Fig. 1C,D). This increased expression of retrotransposons was accompanied by increased γ‐H2AvD foci in the nuclei of fat bodies depleted of *AGO2* (Fig. 2A,F,H). Therefore, retrotransposon up‐regulation in fat bodies may lead to increased DNA damage.

Next, we asked whether DNA damage could be reduced in old fat bodies by repressing retrotransposon expression. We utilized an *AGO2* EP line (Bellen *et al*., 2004) that allowed *Cg‐Gal4*‐induced overexpression of *AGO2* in fat bodies. qRT‐ PCR confirmed an elevated expression of *AGO2* (Fig. S1). *AGO2* elevation resulted in a noticeable decrease of the number of nuclear γ‐H2AvD foci in the old fat bodies (compare Fig. 2G to 2C). Our quantification using randomly selected nuclei showed a small but significant decrease of the average nuclear γ‐H2AvD intensity in these old fat bodies (Fig. 2H). These findings suggest that increased expression of retrotransposons could contribute toward the increased DNA damage in old fat bodies.

### LAM may repress the expression of multiple retrotransposons in fat bodies

**Figure 3:**
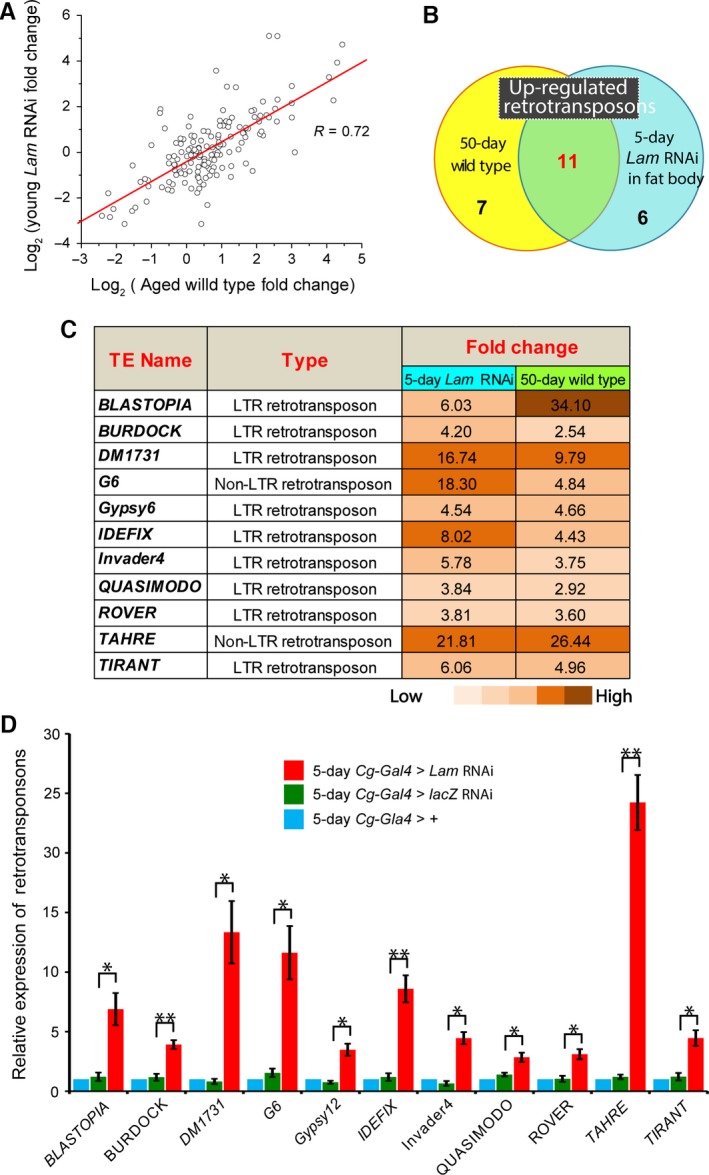
De‐repression of retrotransposons in fat bodies upon fat body‐specific LAM depletion. (A) Similar changes of retrotransposon expression (compared to the 5‐day fat bodies) in old fat bodies or young fat bodies depleted of LAM. The retrotransposons that exhibited altered expression upon aging or LAM depletion were compared with one another. R, Pearson correlation coefficient. (B) Pie chart showing 11 identical de‐repressed (by more than 2 fold) retrotransposons shared between the old fat bodies and LAM‐depleted young fat bodies. (C) A list of up‐regulated retrotransposons (by more than 2 fold) found upon aging and upon LAM depletion in young fat bodies (compared to the control 5‐day old fat bodies). The numbers and color codes show the fold of upregulation. (D) qRT‐PCR analyses of the indicated retrotransposons found to be upregulated by RNA‐seq in young (5 day) fat bodies depleted of LAM (*Cg‐gal4/+; tub‐gal80* ^*ts*^ */Lam RNAi*). The qRT‐PCR analyses showed that the increased expression of the indicated retrotransposons in the LAM‐depleted young fat bodies (*Cg‐gal4/+; tub‐gal80* ^*ts*^ */Lam RNAi*) were all significantly up‐regulated as compared to the control young fat bodies depleted of *lacZ* (*Cg‐gal4/+; tub‐gal80* ^*ts*^ */ lacZ RNAi*). The fold expression change was plotted relative to 5‐day‐old control fat bodies (*Cg‐gal4/+; tub‐gal80* ^*ts*^ */+*), which was set to 1. Error bars, SEM based on three independent experiments. Student's t test: *P < 0.05, **P < 0.01.

Our previous studies have shown that *Drosophila* aging is accompanied by a gradual reduction of lamin‐B, LAM (encoded by the gene *Lam*) (Chen *et al*., 2014). Since LAM is involved in maintaining heterochromatin, its loss could contribute to the age‐ associated increase in retrotransposon expression and DNA damage in fat bodies. We therefore asked whether LAM depletion in young fat bodies could lead to a similar up‐regulation of retrotransposons as seen in old fat bodies. The global expression profiles of retrotransposons appeared similar between old fat bodies from wild‐type flies and young fat bodies depleted of LAM (*Cg‐gal4/+; tub‐Gal80* ^*ts*^ */Lam RNAi*) (Fig. 3A).

By comparing the retrotransposon expression profiles between 5‐day old control fat bodies (*Cg‐Gal4/+; tub‐Gal80* ^*ts*^ */+*) and the 5‐day old flies with LAM depleted in fat bodies, we found that 17 retrotransposons were up‐regulated at least 2‐fold upon LAM depletion (Table S2). Importantly, 11 of these 17 de‐repressed retrotransposons were also found to be de‐repressed in wild‐type fat bodies from old flies (Fig. 3B). Additionally, most (64.7%) of these de‐repressed retrotransposons exhibited similar degrees of up‐regulation in both the young fat bodies depleted of LAM and the old fat bodies (Fig. 3C). qRT‐PCR analyses further confirmed that LAM depletion in young fat bodies resulted in increased retrotransposon expression, whereas control depletion by LacZ RNAi or GFP RNAi in fat bodies had no effect (Fig. 3D and Fig. S2). This suggests that LAM could play a role in repressing the retrotransposons in the fat bodies.

### Age‐associated LAM loss may contribute to the formation of DNA damage foci in the nuclei of fat body cells

**Figure 4:**
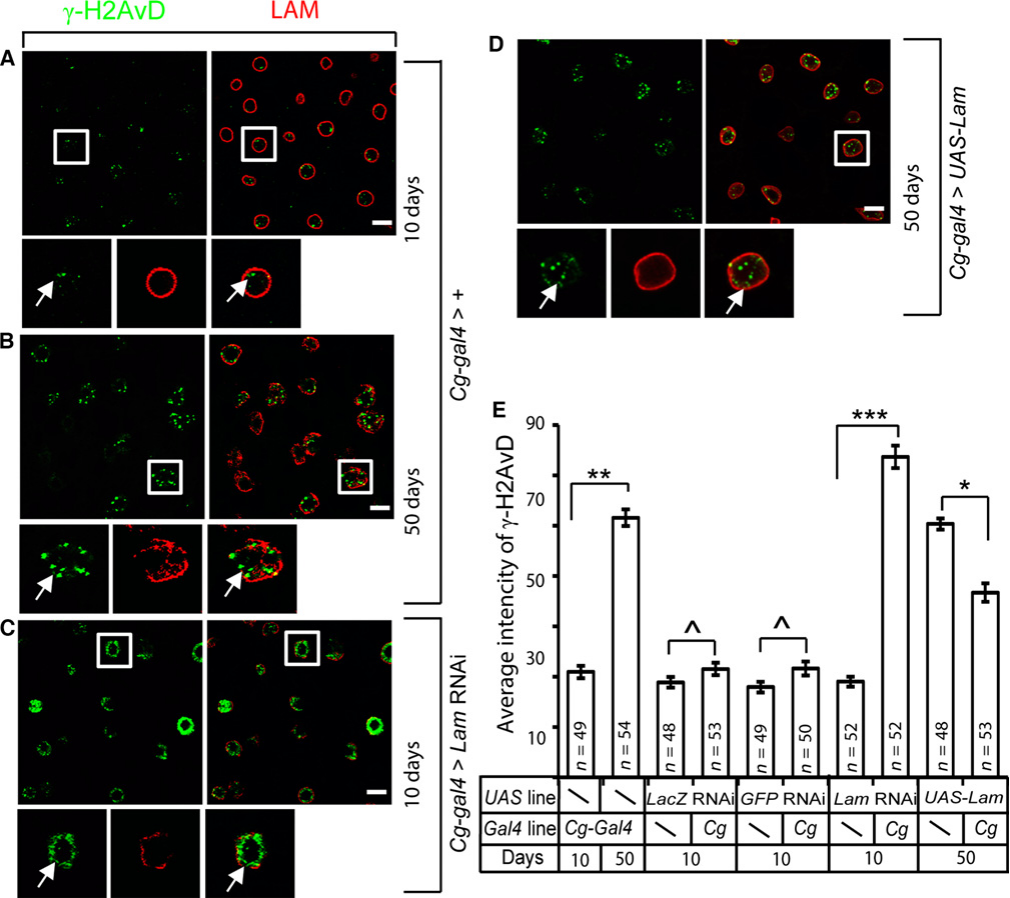
Age‐associated LAM loss may contribute to DNA damage in the fat bodies. (A–D) Compared to control young fat bodies (A, *Cg‐gal4/+; tub‐gal80* ^*ts*^ */+*), increase in γ‐H2AvD signal (green) in old fat bodies (B) could be mimicked by fat body‐specific depletion of LAM (red) in young flies (C, *Cg‐gal4/+; tub‐gal80* ^*ts*^ */Lam RNAi*), whereas forced expression of LAM in old fat bodies (D, *Cg‐gal4/+; tub‐gal80* ^*ts*^ */UAS‐Lam)* significantly reduced γ‐H2AvD signal. Nuclei boxed by white squares are enlarged to the bottom. Images were all taken from the A4 region. White arrows indicate γ‐H2AvD staining foci. Scale bars, 20 μm. (E) Quantification of γ‐H2AvD intensities in fat body cells from 10 or 50‐day‐old Gal4 control flies (*Cg‐gal4/+; tub‐gal80* ^*ts*^ */+*), UAS control flies (for *Lam RNAi, LacZ RNAi, GFP RNAi,* and *UAS‐Lam*), 10‐day‐old flies carrying *Cg‐gal4/+;tub‐ gal80* ^*ts*^ */Lam RNAi, Cg‐gal4/ LacZ RNAi;tub‐gal80* ^*ts*^ */+,* or *Cg‐Gal4/+;tub‐gal80* ^*ts*^ */GFP RNAi*, and 50‐day‐old flies carrying *Cg‐Gal4/+;tub‐gal80* ^*ts*^ */UAS‐Lam*. The γ‐H2AvD signal was determined by measuring the total nuclear immunofluorescence signal from the images captured using the same microscopy settings (see the detailed description in the experimental procedures). Nuclei from the A4 region (see Fig. 1A) were measured to allow the comparison among different fat bodies. Error bars, SEM. Student's t‐test: ^*P *> 0.5, *P < 0.5, **P < 0.01, ***<0.001. The numbers (*n*) of fat body cells analyzed shown were from three independent experiments.

Since up‐regulation of retrotransposons in the fat body resulted in increased DNA damage foci formation in aging flies and since LAM depletion in young fat bodies resulted in retrotransposon de‐repression, we asked whether LAM depletion in young fat bodies could lead to increased DNA damage foci formation. Compared to young fat bodies from control flies (*Cg‐Gal4/+;tub‐Gal80* ^*ts*^ */+*, Fig. 4A,E), we found that LAM‐depleted young fat bodies (*Cg‐gal4/+;tub‐Gal80* ^*ts*^ */Lam RNAi*) exhibited a significant increase of nuclear γ‐H2AvD foci (Fig. 4C,E). The degree of increase by LAM depletion was similar to those observed in the 50‐day old control fat bodies (*Cg‐gal4/+;tub‐Gal80ts/+*, Fig. 4B,E), whereas the control RNAi depletion of LacZ or GFP in fat bodies had no effect (Fig. 3E). Next, we asked whether expressing LAM in old fat bodies could reduce the DNA damage foci. LAM was expressed in the old fat body using the Gal4/Gal80^ts^‐UAS system. We found that induced expression of LAM in old fat bodies (*Cg‐gal4/+;tub‐Gal80* ^*ts*^ */UAS‐Lam*) resulted in noticeable decrease in the number and intensity of the nuclear γ‐H2AvD foci in at least some nuclei compared to controls (compare Fig. 4D to 4B). To confirm this, we quantified the total nuclear γ‐H2AvD intensity in randomly selected nuclei and found a small but significant reduction upon LAM expression in old fat bodies (Fig. 4E). These findings suggest that LAM reduction in aging fat bodies could contribute to the elevated DNA damage foci formation in the nuclei of this tissue.

### LAM contributes to the maintenance of histone H3 lysine 9 trimethylation in the fat body

Previous studies show that retrotransposons are regulated by both chromatin structure and RNAi machinery. Since Dicer2‐AGO2‐mediated endogenous small interfering RNA (esiRNAs) pathway regulates the expression of TEs in *Drosophila* somatic cells, we examined whether *Dicer‐2* and *AGO2* undergo age‐associated expression change. Both RNA‐seq and RT‐qPCR revealed similar expression of *Dicer‐2* and *AGO2* in young and old fat bodies (Fig. S3A,B). Therefore, retrotransposon de‐repression upon natural aging or upon LAM depletion in fat body is not due to decreased expression of the two proteins required for the esiRNA pathway.

**Figure 5:**
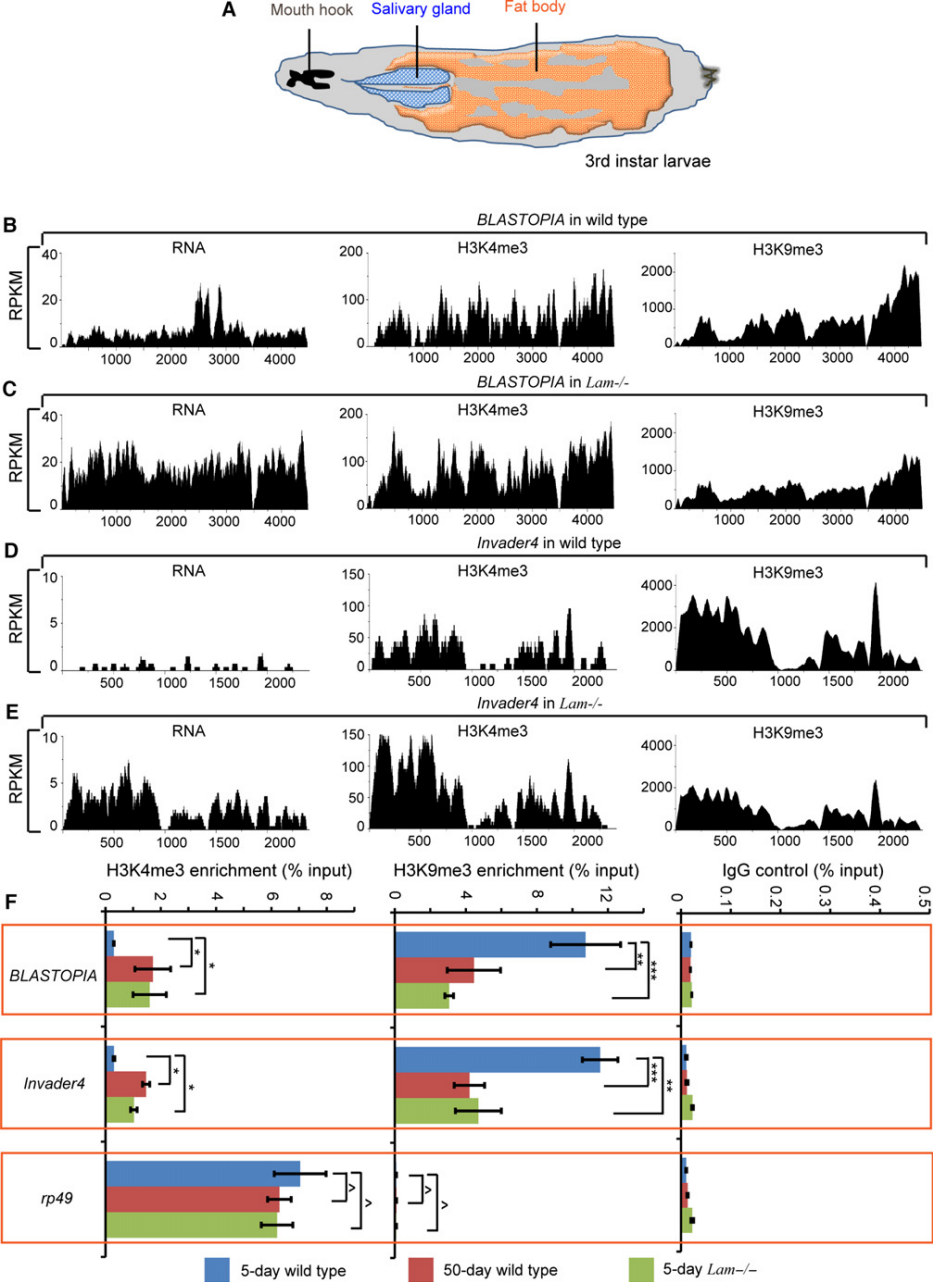
LAM inhibits retrotransposon expression by maintaining heterochromatin. (A) A cartoon illustration of the third instar larvae. Fat body (orange) aligns the inner cuticle surface of most of the larval body. The mouth hook (black) and salivary gland (blue) are also shown. (B–E) RPKM (Reads Per Kilobase per Million mapped reads) values of RNA expression, H3K4me3, and H3K9me3 modification for the indicated two retrotransposons (*BLASTOPIA* and *Invader4*) in the wild‐type and *Lam* ^*−/−*^ (*Lam* ^*D395*^ */Lam* ^*k2*^) third instar fat bodies. Increased retrotransposon expression was accompanied by decreased H3K9me3 and increased H3K4me3 modifications. (F) Chromatin immunoprecipitation (ChIP)‐qPCR analysis of selected retrotransposons (*BLASTOPIA* and *Invader4*) of fat body dissected from the 5‐day or 50‐day wild‐type (*w* ^*1118*^), or 5‐day *Lam* ^*−/−*^ (*Lam* ^*D395*^ */Lam* ^*k2*^) flies. Chromatin was immunoprecipitated with antibodies to H3K4me3, H3K9me3, or control IgG. Primers corresponding to *BLASTOPIA*,* Invader4*, and rp49 (control) were used to amplify precipitated DNA. ChIP samples were normalized to the input DNA. Error bars, SEM, based on three independent experiments. Student's *t*‐tests: ^*P *> 0.05, *P < 0.05, **P < 0.01.

Since LAM could contribute toward heterochromatin maintenance by binding to chromatin regulators such as HP1, we wish to determine whether LAM reduction in old fat bodies might contributes to retrotransposon de‐repression by reducing the histone H3 lysince 9 trimethylation (H3K9me3) modification. Unfortunately, it is very difficult to obtain enough pure fat body cells from adult flies for genome‐wide analyses based on chromatin immunoprecipitation sequencing (ChIP‐seq). On the other hand, one can obtain relatively large quantities of pure third instar larva (L3) fat bodies (Fig. 5A) for ChIP‐seq. Since *Lam* ^*−/−*^ (*Lam* ^*D395*^ */Lam* ^*k2*^) flies could survive to third instar larvae stage, we first analyzed whether the *Lam* ^*−/−*^ fat bodies exhibited retrotransposon de‐repression by RNA‐seq. We identified 51 up‐regulated retrotransposons (fold change>2, FDR < 0.05) in *Lam* ^*−/−*^ larvae fat bodies compared to those of the wild‐type (Table 1). Among these up‐regulated retrotransposons, 14 were also up‐regulated either in the old fat bodies or in the LAM‐depleted young fat bodies.

**Table 1:**
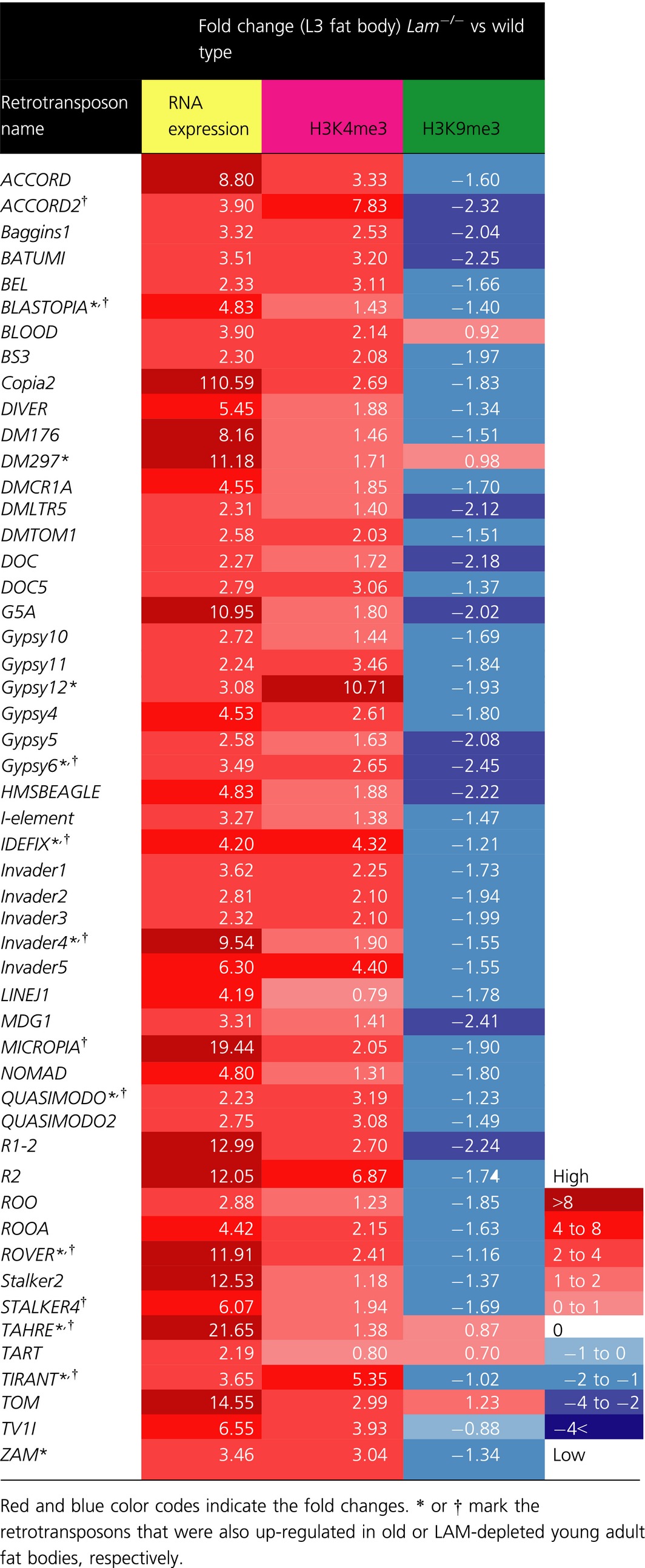
A list of retrotransposons whose expressions are increased in the *Lam* ^*−/−*^ (*Lam^D395^/Lam^k2^*) L3 larvae along with the changes of H3K4me3 and H3K9me3

Next, we dissected fat bodies from wild‐type and *Lam* ^*−/−*^ third instar larvae and performed H3K4me3 or H3K9me3 ChIP‐seq. We found a significant decrease of H3K9me3 levels in the chromatin regions containing the de‐repressed retrotransposons, whereas the H3K4me3 was increased in these regions for most retrotransposons. This finding can be demonstrated by plotting the log2 fold change of H3K4me3 or H3K9me3 for each retrotransposons on the *y*‐axis using the box plot (Fig. S3C), which shows that upon LAM depletion, most retrotransposons exhibit H3K4me3 increase (>0 on *y*‐axis) and H3K9me3 decrease (<0 on *y*‐axis). By analyzing H3K9me3 and H3K4me3 levels on the de‐repressed retrotransposons, we found that the de‐repression is accompanied by a significant increase and decrease of H3K4me3 and H3K9me3, respectively (Table 1). We further confirmed these findings by ChIP‐qPCR analyses of four retrotransposons selected form Table 1 using dissected fat bodies from wild‐type and *Lam* ^*−/−*^ third instar larvae (Fig. S4A). Moreover, we present the genome browser views for retrotransposon RNA, H3K4me3, and H3K9me3 reads for two de‐repressed retrotransposons, *BLASTOPIA* and *Invader4*, obtained from the wild‐type and *Lam* ^*−/−*^ L3 larvae (Fig. 5B–E).

Although it is difficult to obtain enough pure fat bodies from old flies for ChIP‐seq, we succeeded to use ChIP‐qPCR to study H3K9me3 and H3K4me3 modifications of four selected de‐repressed retrotransposons (*BLASTOPIA*,* Gypsy6*,* Invader,4* and *QUASIMODO*) due to aging or LAM deletion in the dissected control young or old wild‐type fat bodies and the LAM‐depleted young fat bodies. We found that these retrotransposons indeed exhibited decreased H3K9me3 and increased H3K4me3 upon aging or upon LAM depletion (Fig. 5F and Fig. S4B). Taken together, our findings suggest that LAM reduction upon aging could contribute to the loss of heterochromatin and retrotransposon activation in the fat bodies.

## Discussion

**Figure 6:**
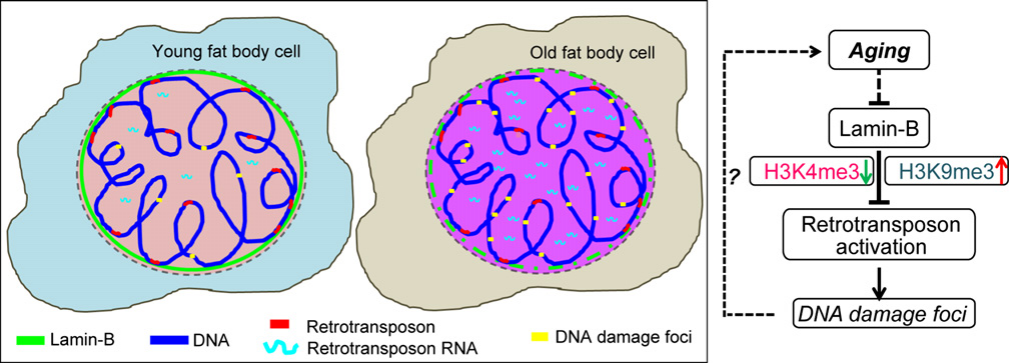
A model. Age‐associated lamin‐B reduction in fat body may lead to a reduction of heterochromatin and de‐repression of retrotransposons, which could in turn contribute to an increased retrotransposition and DNA damage in this organ.

By analyzing a few selected retrotransposons, previous studies have shown that organismal aging can lead to the activation of retrotransposons in several tissues. There has been, however, no report on genome‐wide analyses of retrotransposon de‐repression in dissected old tissues thus far. Here we report that ~16% of retrotransposons undergo over 2‐fold increase in expression in the old *Drosophila* fat bodies. Our analyses suggest the possibility that retrotransposon up‐regulation could contribute to the increased formation of DNA damage foci in this *Drosophila* tissue. By analyzing the *Lam* ^*−/−*^ fat bodies from the third instar larvae, the LAM‐depleted young fat bodies, and the old fat bodies known to exhibit LAM reduction, our findings suggest that LAM loss could contribute to the loss of heterochromatin‐based repression of retrotransposons. Based on our findings, we propose a speculative model that increased formation of DNA damage foci in the old fat body, caused in part by the increased retrotransposon expression as a result of LAM reduction, contribute to fly aging phenotypes (Fig. 6). It will be important to further establish whether and how LAM directly contributes to the establishment and maintenance of the heterochromatin in the fat body. Further studies will be required to determine whether the increased retrotransposon expression is accompanied by increased retrotransposition in the fat body.

Previously, we have shown that age‐associated LAM reduction in fat bodies leads to increased systemic inflammation, which contributes to gut hyperplasia in old flies (Chen *et al*., 2014). Repression of activities of the immune deficiency (IMD) pathway in old fat bodies can only partially reverse the hyperplastic phenotype in the gut. While the partial rescue could be due to insufficient RNAi‐mediated silencing, it is also possible that the age‐associated LAM reduction in the fat body could contribute to gut hyperplasia due to increased DNA damage in fat body cells. Interestingly, DNA damage is known to trigger alterations of metabolism in cells (Shimizu *et al*., 2014). As the major organ that controls sugar and lipid metabolism, increased DNA damage in fat bodies may contribute to gut hyperplasia independent of the inflammatory pathways. Despite the potential connection between DNA damage and disease, we stress that the increased DNA damage foci formation as revealed by the γ‐H2AvD staining may not reflect an actual increase in double strand DNA breaks. Therefore, it will be important to further explore whether the age‐ associated increase in retrotransposon expression in fat bodies indeed leads to increased DNA damage, how the DNA damage may contribute to the metabolic dysfunction of this organ, and how this may in turn influence the gut and other organs.

The declined ability to maintain heterochromatin has been established as one of the major culprit for aging phenotypes. A number of studies have shown that heterochromatin loss can lead to the activation of TEs. Age‐associated decline of histones or chromatin regulators known to control the heterochromatin state have been shown to contribute to the loss of heterochromatin and TE activation. More recently, DICER1 decline in aging retina has been linked to *Alu* retrotranspon activation and macular degeneration (Tarallo *et al*., 2012). Our studies add another protein, lamin‐B, into the list of proteins whose age‐associated decline may contribute to retrotransposon activation. It will be important to further dissect how these proteins work together to maintain the heterochromatin‐mediated silencing of TE.

Additionally, it is not clear why aging is associated with the decline of some proteins but not the others. It is possible that the decline of one of these nuclear proteins leads to the reduction of other nuclear and cytoplasmic proteins through transcriptional regulation. Alternatively, different pathways may regulate the decline of different nuclear proteins. Finally, it is unclear whether the age‐associated decline in protein levels as detected by Western blotting or immunostaining is due to protein reduction or protein modification change, as altered modifications may not only block antibody recognition but could also result in a dysfunctional protein. Research toward addressing how aging leads to the reduction of proteins or protein functions required for maintaining heterochromatin will help to answer these questions.

## Experimental procedures

### Fly stocks, genetic manipulations, and culture condition

We used *w* ^*1118*^ (Bloomington Stock Center #3605) as the wild‐type fly. All other alleles were backcrossed into *w* ^*1118*^ flies for at least five generations. LAM lines: *Lam* ^*D395*^ (null) (Munoz‐Alarcon *et al*., 2007), *Lam* ^*k2*^ (LAM truncation, Bloomington Stock Center #25093) (Patterson *et al*., 2004), *Lam* ^*D395*^ */Lam* ^*k2*^ transheterozygous (as *Lam* ^*−/−*^), and *Lam* cDNA transgenic line (*UAS‐Lam* ^*354*^) (Munoz‐Alarcon *et al*., 2007). RNAi alleles: *Lam RNAi* (Vienna *Drosophila* Resource Center, #v45635), *AGO2 RNAi* (Bloomington Stock Center, #34799 and #55672), *LacZ RNAi* (from Allan Spradling), and *GFP RNAi* (Bloomington Stock Center, #9330). UAS alleles: *AGO2* ^*EY04479*^ (Bloomington Stock Center, #16608). Gal4 alleles used to drive UAS lines: *Cg‐Gal4* (Bloomington stock center # 7011) for fat body. The *tub‐Gal80* ^*ts*^ alleles (Bloomington Stock Center #7018 and #7019) were used to control Gal4 expression.

All flies were maintained on the standard cornmeal/molasses/yeast fly food (the recipe for 1 L food is: cornmeal 50 g, yeast 18.75 g, agar 5 g, molasses 62.5 mL, 5% anti‐fungal agent Tegosept 11.5 mL, and propionic acid 35 mL) and cultured at 25 °C in a normal light/dark cycle, unless noted otherwise. To deplete LAM, LacZ or GFP in fat body by Gal4‐mediated RNAi in 5‐ or 10‐day old flies, *Cg‐Gal4;tub‐ Gal80* ^*ts*^ was used to drive the RNAi alleles. The crosses were maintained at 19 °C to repress the UAS‐GAL4 system. One day or five days after eclosion, the adult were shifted to 29 °C to turn on the UAS‐Gal4 system, which induces RNAi. These flies were then incubated at 29 °C for 5 days followed by immunostaining, qRT‐PCR, RNA‐seq, or Western blotting analyses. The Gal4 lines and UAS lines, which were treated in the same way in parallel, were used as controls.

To inhibit AGO2 expression in fat body of 5‐day old flies, *Cg‐Gal4;tub‐Gal80* ^*ts*^ was used to drive *AGO2* shRNA alleles. The crosses were maintained at 19 °C to repress the UAS‐Gal4 system. One day before eclosion, the pharate adult were shifted to 29 °C and the eclosed flies were maintained at 29 °C for 5 followed by dissection of fat bodies for immunostaining or qRT‐PCR. The Gal4 lines (Gal4s with *tub‐Gal80* ^*ts*^) and UAS lines, which were treated in the same way in parallel, were used as controls.

To rescue aging phenotype by ectopic LAM or AGO2 expression in fat body, we used *Cg‐Gal4;tub‐Gal80* ^*ts*^ *t*o drive *UAS‐Lam* cDNA or AGO2 EP line, respectively. The crosses were performed at 19 °C and shifted to 25 °C one day before eclosion. To induce LAM or AGO2 expression in adult fat body, we shifted the flies from 25 °C to 29 °C for 24 h on days 15, 25, 35, and 45. The fat bodies were then dissected on day 50 and processed for immunostaining. The Gal4 lines (Gal4s with *tub‐Gal80* ^*ts*^) and UAS lines, which were treated in the same way in parallel, were used as controls.

### Adult fat body dissection, qRT‐PCR, RNA‐seq and analyses

Fat body dissection, RNA isolation, qRT‐PCR, RNA‐seq and analyses were described previously (Chen *et al*., 2014).

qRT‐PCR primers

*Blastopia*:
  Forward primer (F): GAGCAGTCAATCGTCCGTAA
  Reverse primer (R): TCTATAGTCCACGCAAACGC
*Burdock*:
  F: GGACGCGTTACGTGTATTTG
  R: ATAAGGGCGAATTGGTAACG
*ADO2*:
  F: ATTACCATTACCGCCTCAGC
  R: GGACAGGTCAAGGTCCAGAT
*Dicer‐2*:
  F: ACTATGTGCGCATTCTGCTC
  R: TGGGACTCCTTTCTCTGCTT
*DM1731*:
  F: GAGAAATCACTTTGGGCCAT
  R: TCGTCGCTGGTCTACAGTTC
*G6*:
  F: AAAGGACTCCACCACTCCAC
  R: GTTCTTCTGCCAATGGGATT
*Gypsy6*:
  F: GTTATACCCGTACCCGATGG
  R: GGTCATCGGTCCCTTTCTTA
*Gypsy12*:
  F: TGACTCGGCTGATGTTTCTC
  R: GAAACACAGGTGGAATCGTG
*IDEFIX*:
  F: CGCTCTAGTGGACAGAACCA
  R: TGAAATGAGGCATTTGGGTA
*Invader4*:
  F: TCCGATCGAGTGGTCATAAA
  R: CGACGTCAGCAGTCAAACTT
*IVK*:
  F: ACTCTGGGTTCCCAGTCATC
  R: GGTCCTTGGAGTTAAACGGA
*QUASIMODO*:
  F: TCTACAGTGCCATCGAGAGG
  R: TAGTTCAGCCCAAGTGTTGC
*ROVER*:
  F: CAGACCCAACTCAAATCCCT
  R: CGTTTGGTGCTGTCTGTTCT
*TAHRE*:
  F: ATCCAGGCCAAGGATATGAC
  R: TCTGATGATGACTCGGAAGC
*TIRANT*:
  F: GCAATGCCAAATAGAGCAGA
  R: TACGGTCATTTCGGTCGTTA
*ZAM*:
  F: GATGGTACTCCAGGCCAACT
  R: AGAGCTTCGTCTGCTCTTCC
*rp49*:
  F: TACAGGCCCAAGATCGTGAA
  R: TCTCCTTGCGCTTCTTGGA

### Immunofluorescence microscopy and quantifications

Adult fat bodies with attached cuticles were dissected in PBs. The details of the staining procedure have been described previously (Chen *et al*., 2014). All primary antibodies were preabsorbed using embryos fixed by 4% paraformaldehyde. The following primary antibodies were used: rabbit anti‐Histone H2AvD pS137 (anti‐γ‐ H2AvD, Rockland #600‐401‐914, 1:5000), mouse anti‐LAM (DSHB #ADL67, 1:100). The secondary antibodies used, Alexa 488 and Alexa 568 from Molecular Probes/Invitrogen (1:1000). DNA was stained with 4,6‐diamidino‐2‐phenylindole (DAPI; Sigma) at 1 μg/mL.

A Leica TCS‐SP5 confocal microscope was used to acquire all immunofluorescence images. For each set of experiments, images were acquired as confocal stacks using the same setting. Adobe Photoshop and Adobe Illustrator CS3 were used to assemble the images.

To quantify the nuclear immunofluorescence intensity of γ‐H2AvD, the fat body nuclei were imaged using an Epitome microscope (Zeiss ApoTome.2). We used the same method as described in our previous paper (Wan *et al*., 2015) to determine the nuclear fluorescence signal from the epifluorescence images. Briefly, we defined the nuclear regions by drawing a circle X based on the DAPI staining. Then, we draw a larger circle around the nucleus as Y. The areas of X and Y were determined as Xa and Ya. The mean fluorescence intensity for X and Y were measured as Xf and Yf.

The signal intensity for each nucleus was defined ad Xi and was determined as Xi = Xf − (Yf × Ya – Xf × Xa)/(Ya − Xa). All experiments were performed three times. About 50 nuclei were quantified from these experiments for each genotype and plotted.

ChIP‐seq analyses and additional detailed descriptions of all methods can be found in the Supporting information.

## Funding

No funding information provided.

## Conflict of interest

None declared.

## Author contributions

Haiyang Chen designed and performed all experiments, Xiaobin Zheng analyzed all the RNA‐seq and ChIP‐seq data, Haiyang Chen and Yixian Zheng conceived the project, interpreted the data, and wrote the manuscript.

## Supporting information


**Fig. S1 (related to Figure 2).** The expression of *AGO2* significantly increased in fat bodies in the *Cg‐gal4‐*driven *AGO2* EP line compared to controls.

**Fig. S2 (related to Figure 3).** The expression of retrotransposons does not significantly change in young fat bodies in the control flies with *Cg‐gal4‐*driven *GFP* RNAi as compared to flies carrying no *GFP* RNAi.

**Fig. S3 (related to Figure 5).** The expression of *Dicer‐2* or *AGO2* did not significantly change either in aged fat bodies or lamin‐B depleted young fat bodies.
**Fig. S4 (related to Figure** 5**).** ChIP‐qPCR analyses of H3K4me3 and H3K9me3 on selected retrotransposons in fat bodies.

**Table S1 (related to Figure 1).** A list of retrotransposons that were down regulated in wild type 50‐day‐old fat bodies as compared to that of the young (5‐day).

**Table S2 (related to Figure 3).** A list of retrotransposons that were up regulated in 5‐day old fat bodies upon LAM depletion.
