# Supplementary figures and images for: Age‐associated de‐repression of retrotransposons in the Drosophila fat body, its potential cause and consequence
Source: Aging Cell. 2016 Apr 12;15(3):542–52. doi: 10.1111/acel.12465 (PMC4854910; doi:10.1111/acel.12465)

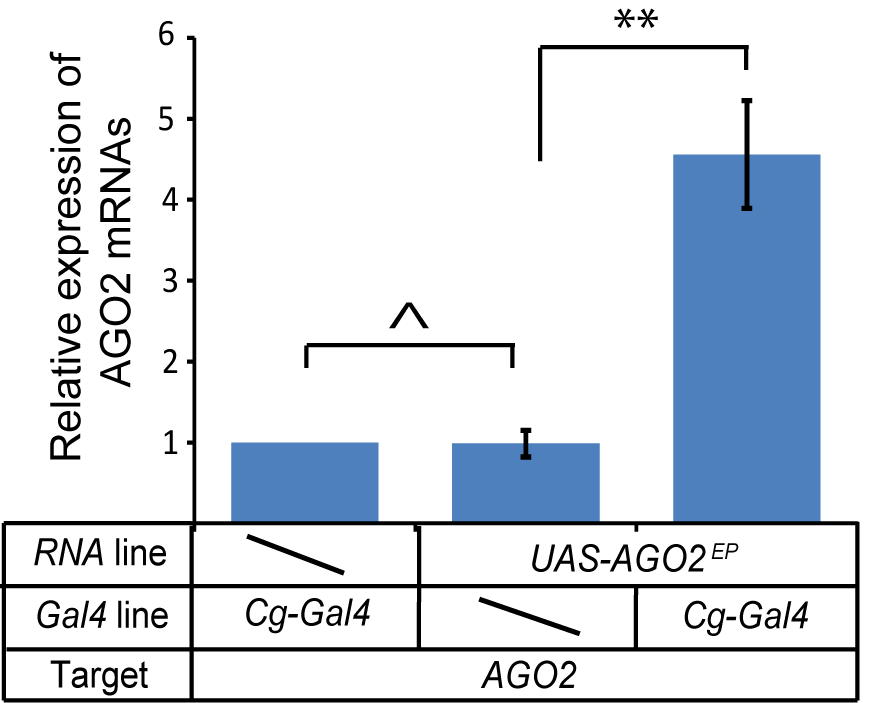

Supplement: Supplementary file 1 — Fig. S1 (related to Figure 2). The expression of AGO2 significantly increased in fat bodies in the Cg‐gal4‐driven AGO2 EP line compared to controls. [file ACEL-15-542-s001.tiff]

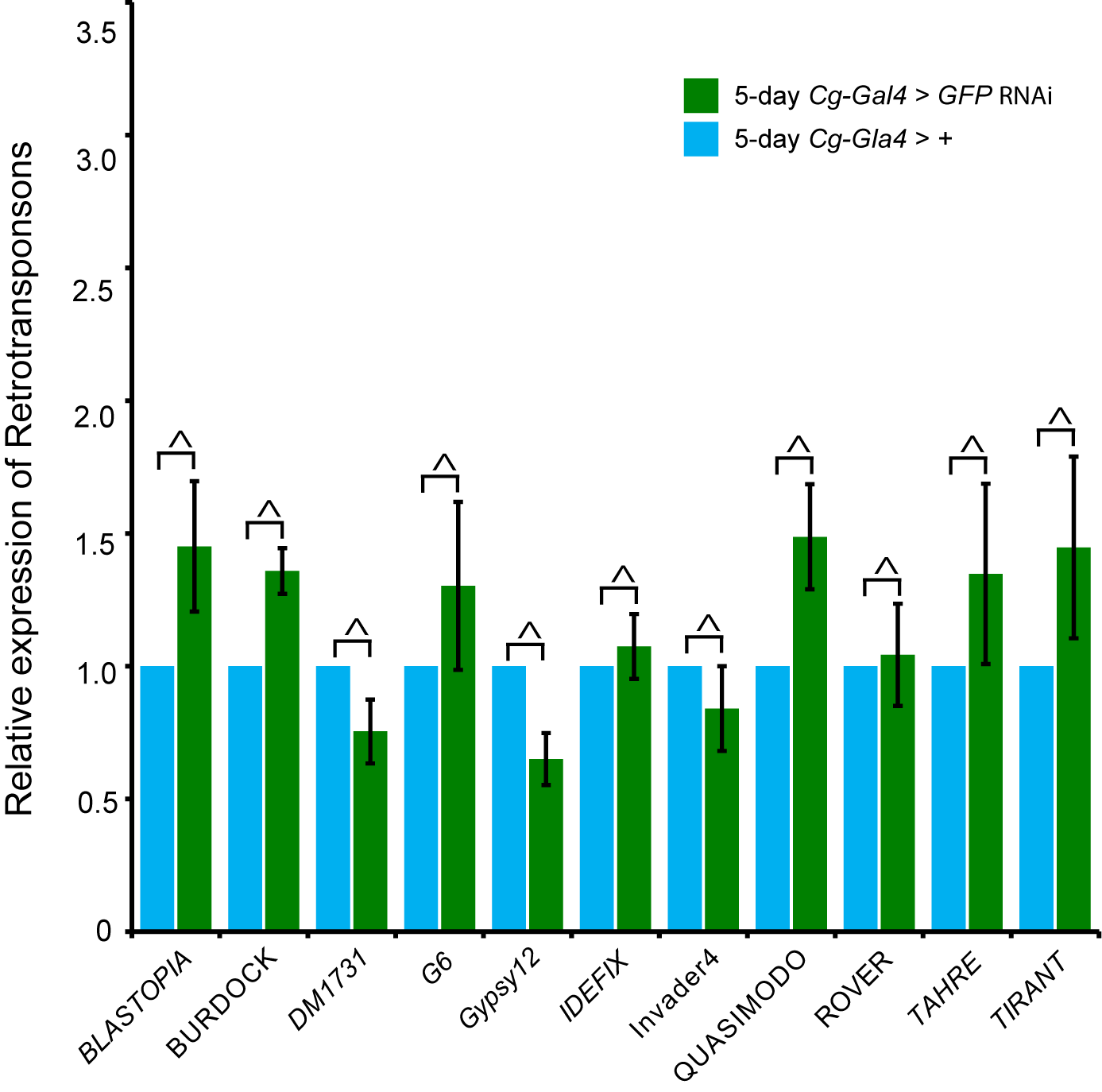

Supplement: Supplementary file 2 — Fig. S2 (related to Figure 3). The expression of retrotransposons does not significantly change in young fat bodies in the control flies with Cg‐gal4‐driven GFP RNAi as compared to flies carrying no GFP RNAi. [file ACEL-15-542-s002.tiff]

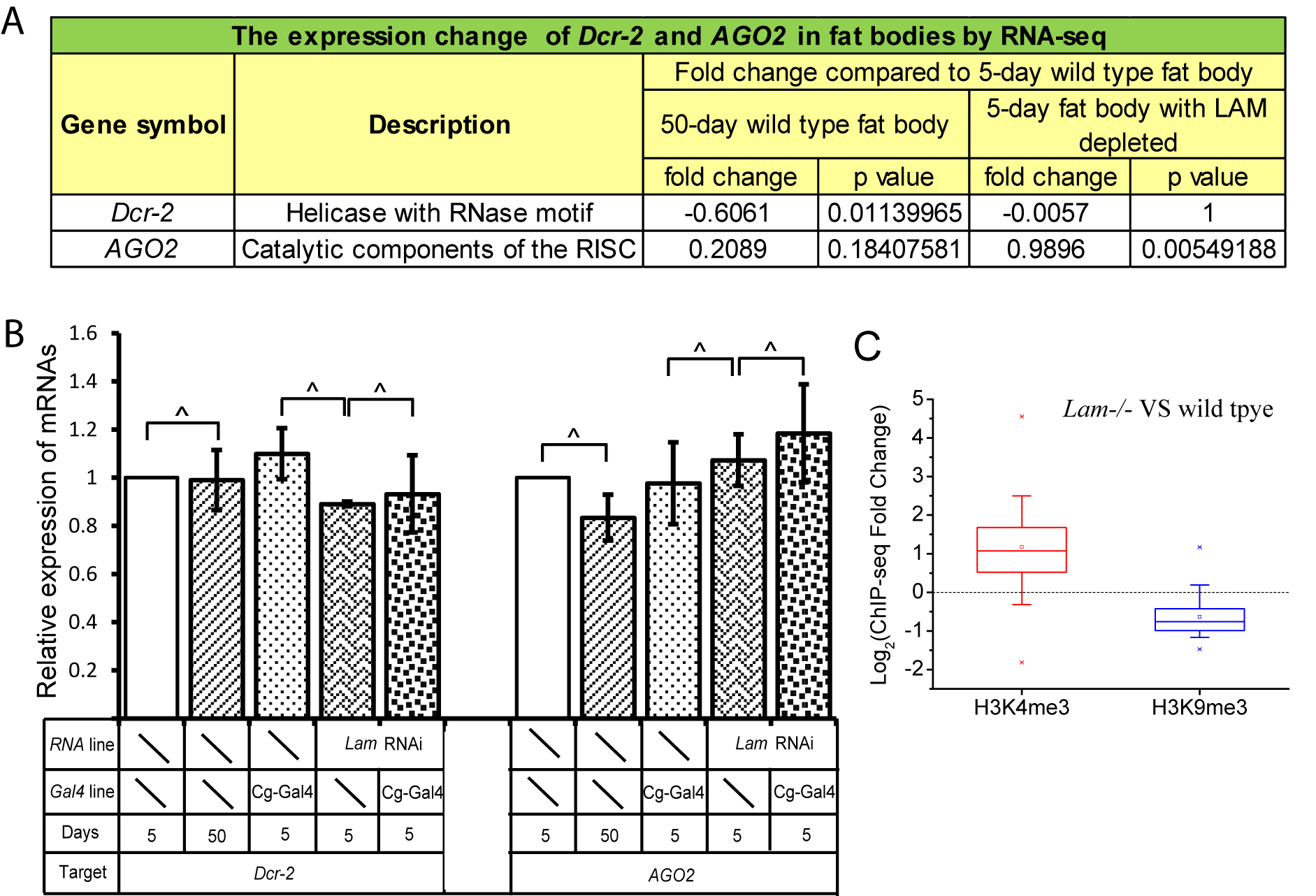

Supplement: Supplementary file 3 — Fig. S3 (related to Figure 5). The expression of Dicer‐2 or AGO2 did not significantly change either in aged fat bodies or lamin‐B depleted young fat bodies. Fig. S4 (related to Figure 5). ChIP‐qPCR analyses of H3K4me3 and H3K9me3 on selected retrotransposons in fat bodies. [file ACEL-15-542-s003.tiff]
