# Supplementary material for: Age‐associated de‐repression of retrotransposons in the Drosophila fat body, its potential cause and consequence
Source: Aging Cell. 2016 Apr 12;15(3):542–52. doi: 10.1111/acel.12465 (PMC4854910; doi:10.1111/acel.12465)
Supplement: Supplementary file 6 [file ACEL-15-542-s006.docx]

**Supplemental Figure and Table Legends**

**Figure S1 (related to Figure 2). The expression of *AGO2* significantly increased in fat bodies in the *Cg-gal4-*driven *AGO2* EP line compared to controls.**

qRT-PCR analyses of *AGO2* in the indicated young (5 day) fat bodies. The expression of *AGO2* is significantly increased in the *AGO2* overexpressing fat bodies (*Cg- gal4/+;tub-gal80ts/UAS-AGO2EP*) compared to the Gal4 (*Cg-gal4/+;tub-gal80ts/+*)

and UAS (*+/+;tub-gal80ts/ UAS-AGO2EP*) controls. The fold expression change was

plotted relative to 5-day-old control fat bodies (*Cg-gal4/+; tub-gal80ts/+*), which was set to 1. Error bars, SEM based on three independent experiments. Student’s t test:

^*p*>0.05, ** *p*<0.01.

**Figure S2 (related to Figure 3). The expression of retrotransposons does not significantly change in young fat bodies in the control flies with *Cg-gal4-*driven *GFP* RNAi as compared to flies carrying no *GFP* RNAi*.***

qRT-PCR analyses of the indicated retrotransposons in young (5 day) fat bodies depleted of GFP (*Cg-gal4/+;tub-gal80ts/GFP RNAi*). The expression of the tested retrotransposons in young fat bodies depleted of GFP (*Cg-gal4/+;tub-gal80ts/GFP RNAi*) does not change compared to the control (*Cg-gal4/+;tub-gal80ts/+*). The fold expression change was plotted relative to 5-day-old control fat bodies (*Cg-gal4/+; tub-gal80ts/+*), which was set to 1. Error bars, SEM based on three independent experiments. Student’s t test: ^*p*>0.05.

**Figure S3 (related to Figure 5). The expression of *Dicer-2* or *AGO2* did not significantly change either in aged fat bodies or lamin-B depleted young fat bodies. A**. RNA-seq showed that the expression of *Dicer-2* and *AGO2* in old fat bodies or LAM depleted young fat bodies was similar to the wild type young fat bodies.

**B**. qRT-PCR analyses of the expression of *Dicer-2* or *AGO2* in wild-type young (5 day) fat bodies, wild-type old (50 day) fat bodies, young Gal4-control fat bodies, young UAS-control fat bodies, or young fat bodies depleted of LAM (*Cg-gal4/+; tub- gal80ts/Lam RNAi*). The fold expression change was plotted relative to 5-day-old

wild-type fat bodies, which was set to 1. Error bars, SEM based on three independent

experiments. Student’s t test: ^*p*>0.05.

**C**. A box-plot showing the distribution of the Log2-fold change for all 111 retrotransposons based on the ChIP-seq of the third instar larval fat bodies from the *Lam-/-* (*LamD395/Lamk2*) and wild type animals. The large boxes in the graph show the

25% and 75% quantiles; the line in each box shows the median; the small square in the middle of each box shows the mean; the upper and lower whiskers show the 90% and 10% quantiles; and the stars show the maximum and minimum values. Thus the plot shows that >75% of all analyzed retrotransposons exhibit increased H3K4me3 and decreased H3K9me3 in lamin-null fat bodies.

**Figure S4 (related to Figure 5). ChIP-qPCR analyses of H3K4me3 and H3K9me3 on selected retrotransposons in fat bodies.**

**A.** ChIP-qPCR analysis of selected retrotransposons (*Accord, BATUMI, Copia2 and Gypsy6*) that become de-repressed upon LAM depletion. Fat bodies were dissected from the third instar larvae of wild type (*w1118*) or *Lam-/-* (*LamD395/Lamk2*) flies. Chromatin was immunoprecipitated with antibodies to H3K4me3, H3K9me3, or control IgG. Primers corresponding to *Accord, BATUMI, Copia2, Gypsy6*, and rp49 (control) were used to amplify the precipitated DNA. ChIP samples were normalized to the input DNA. Error bars, SEM, based on three independent experiments. Student’s t-tests: ^*p*>0.05, **p*<0.05, ***p*<0.01.

**B.** ChIP-qPCR analysis of *Gypsy6* and *QUASIMODO* retrotransposons that become de-repressed upon aging. Fat bodies were dissected from 5-day or 50-day wild type (*w1118*), or 5-day *Lam-/-* (*LamD395/Lamk2*) flies. Chromatin was immunoprecipitated with antibodies to H3K4me3, H3K9me3, or control IgG. Primers corresponding to *Gypsy6*, *QUASIMODO*, and rp49 (control) were used to amplify the precipitated DNA. ChIP samples were normalized to the input DNA. Error bars, SEM, based on three independent experiments. Student’s t-tests: ^*p*>0.05, **p*<0.05, ***p*<0.01.

**Table S1 (related to Figure 1). A list of retrotransposons that were down regulated in wild type 50-day-old fat bodies as compared to that of the young (5-**

**day).**

Retrotransposons, fold changes, p values (by hypergeometric test), and FDR (False

Discover Rate) are indicated.

**Table S2 (related to Figure 3). A list of retrotransposons that were up regulated in 5-day old fat bodies upon LAM depletion.**

Retrotransposon, fold change, P values (by hypergeometric test), and FDR (False

Discover Rate) are indicated.

6 **


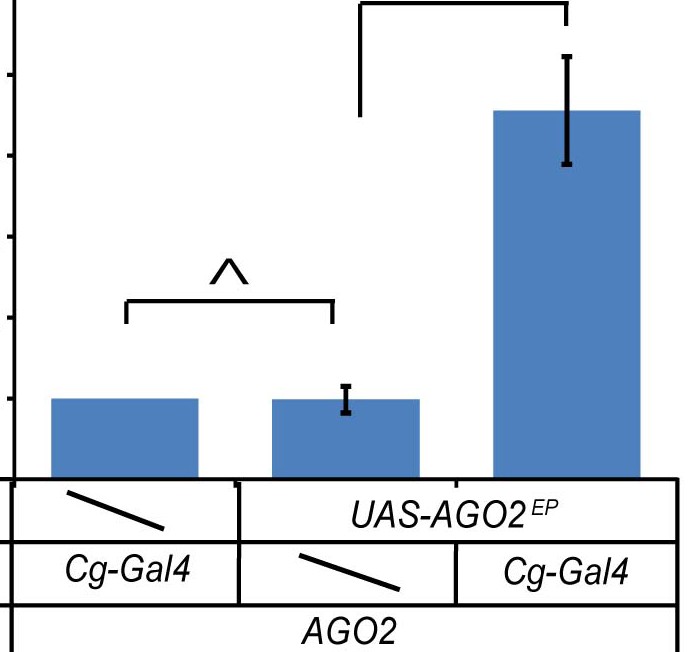


-

0

g (/) 5

·- <(

(1)0::: 4

0..E

XN

3

(1)0 /\

C)

2

<

(

Q)

0::: 1

*RNA* line

Ga/41ine *Cg-Ga/4*

3.5

C/) 3.0

c

0

C/)

c

0

0..

C/) 2.5

c

co

.....

.......

• 5-day *Cg-Ga/4* > *GFP* RNAi

• 5-day *Cg-Gia4* > +

0

.....

.......

0-:::

n*1\ 1\ 1\* n

Q) 2.0 *1\*


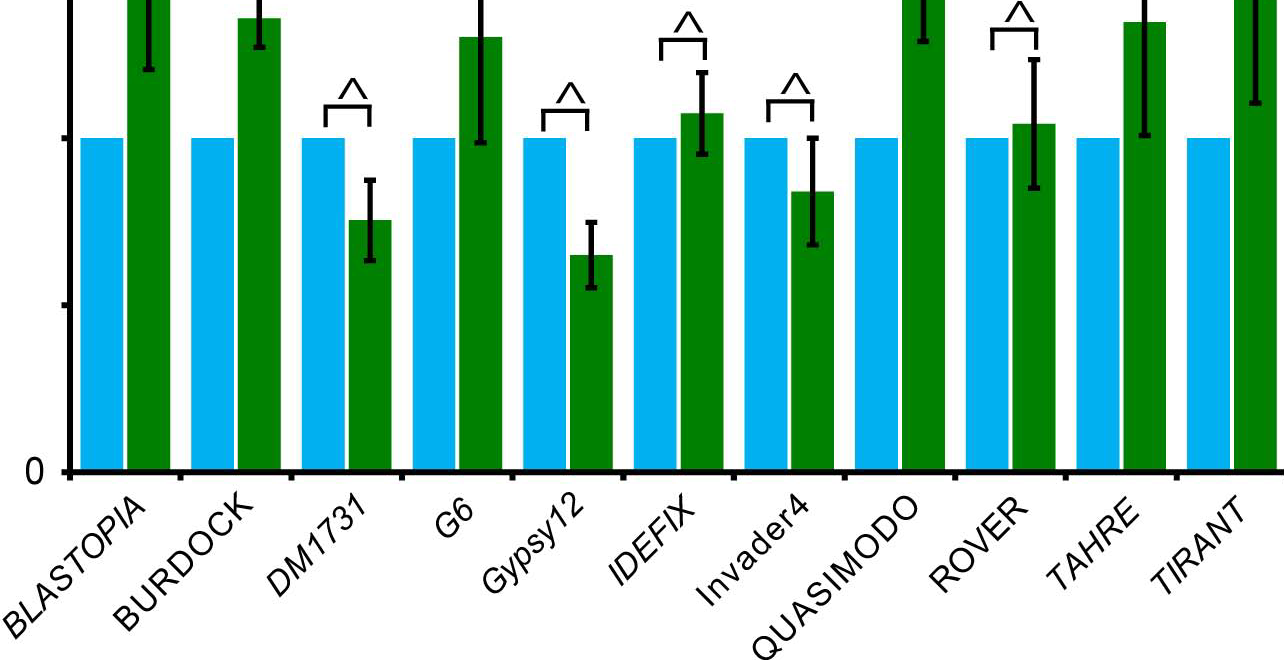


0 n*1\* n n

c *1\*

0 n

1.5

*·c:n*

C/)

Q....).

0..

X

Q) 1.0

Q)

:;>:::;

co

Q)

0::: 0.5

**A**

| **The expression change of *Dcr-2* and *AG02* in fat bodies by RNA-seq** | | | | | |
| --- | --- | --- | --- | --- | --- |
| **Gene symbol** | **Description** | Fold change compared to 5-day wild type fat body | | | |
|  |  | 50-day wild type fat body | | 5-day fat body with LAM  depleted | |
|  |  | fold change | p value | fold change | p value |
| *Dcr-2* | Helicase with RNase motif | -0.6061 | 0.01139965 | -0.0057 | 1 |
| *AG02* | Catalytic components of the RISC | 0.2089 | 0.18407581 | 0.9896 | 0.00549188 |

**B** *rn* 1.6

<(

z

1.4

0:::

*1\ 1\*

**r--lr--1**

*1\ 1\*

**r--lr--1**

**c**

5

Qj"

*Lam-/-* VS wild tpye

-E 1.2

g' 4

g 1 $0

0

c 1

0

"iii

0.8

*B*"' 3

2

0.6

Q)

0.4

Qi 0.2

0:::

0

-1

*s*

d)

-2

mmmmnmn mnm mmmmmm l mmi mm lmn

---.----------.-----

H3K4me3 H3K9me3


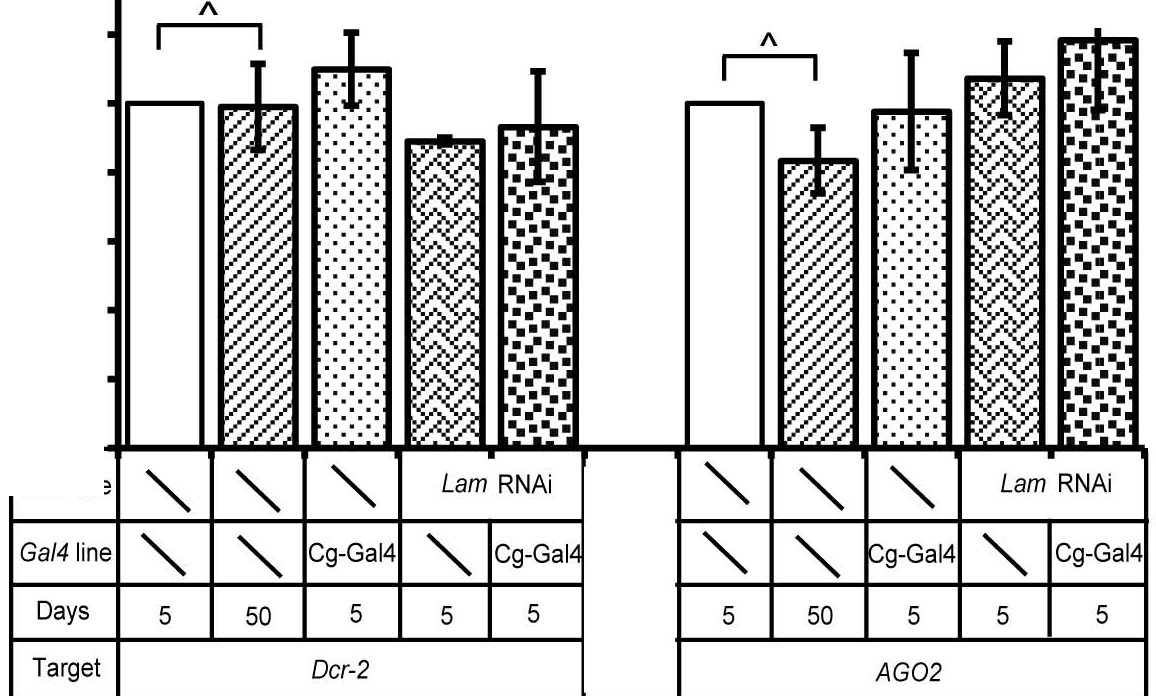


*RNA* line

A H3K4me3 enrichment (% input) H3K9me3 enrichment (% input)

_.


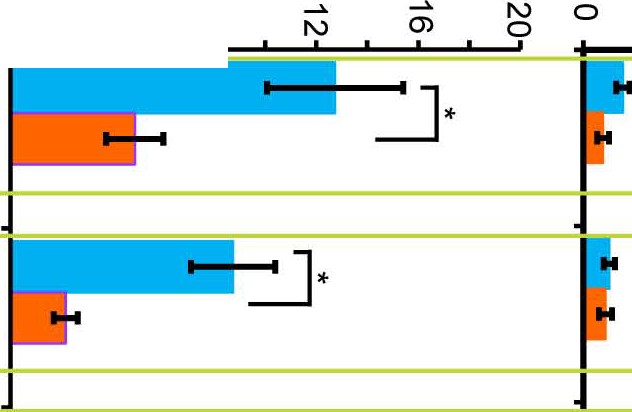

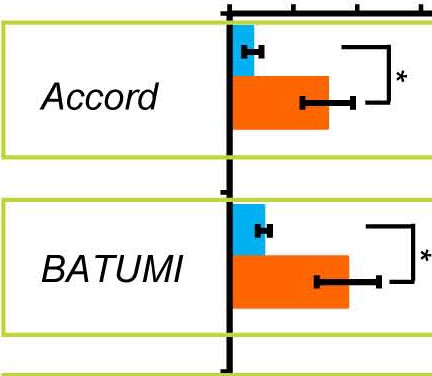


lgG control (% input)

0 <:) <:) 0 0

0 N Ol

CXl 0 CXl Ol

N C..V

()'1

**J·** -+-

=._]:

] **.J**

*Gypsy6* **J· ....,.__j:**

] **J**

]> ***t*** ]>

]

• Third instar larva of wild type • Third instar larva of *Lam-/-*

**B** H3K4me3 enrichment (% input) H3K9me3 enrichment (% input)


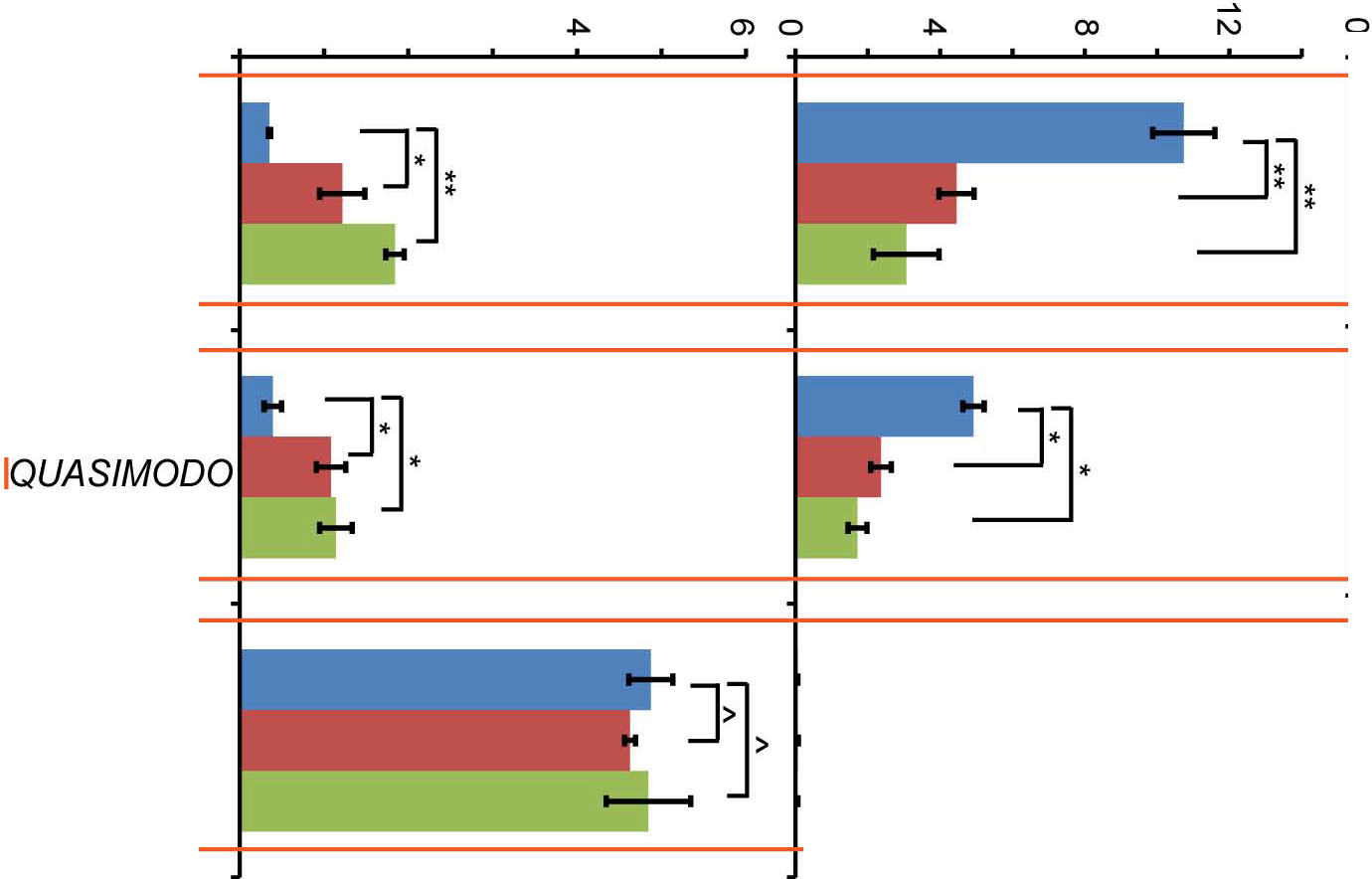

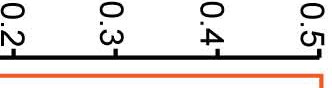


0 N

*Gypsy6*

*rp49*

• 5-day adult of wild type •50-day adult of wild type

lgG control (% input)

0

5-day adult of *Lam-/-*

**A list of retrotransposons that are down regulated in the fat body from**

**50-day-old wild-type flies (compared to 5 day fat body)**

Name of

Retrotransposons fold change P-Value FDR

| ACCORD2 | -2.006832902 | 0.012250251 | 0.043408498 |
| --- | --- | --- | --- |
| Baggins | -3.425975208 | 4.35E-05 | 0.00039385 |
| BLOOD | -3.210349694 | 0.001022757 | 0.006411903 |
| Chimpo | -2.647021164 | 0.004519897 | 0.021668918 |
| DIVER | -3.104897581 | 0.00137382 | 0.007997594 |
| DM176 | -6.911792914 | 1.60E-07 | 2.61E-06 |
| DM412 | -2.343794516 | 0.013512579 | 0.046862773 |
| DMRT1A | -2.669455124 | 0.001257978 | 0.007594459 |
| DMRT1C | -2.448890008 | 0.002827055 | 0.014400312 |
| DOC4 | -2.028518058 | 0.011678733 | 0.042302965 |
| Gypsy7 | -2.10796981 | 0.006899003 | 0.029593091 |
| Invader1 | -8.837317457 | 2.42E-09 | 7.89E-08 |
| MAX | -5.374192232 | 1.83E-06 | 2.29E-05 |
| NINJA | -3.017661594 | 0.000141317 | 0.001151736 |
| NOMAD_I | -2.472522165 | 0.009965241 | 0.037802143 |
| R1 | -8.826819701 | 2.21E-11 | 9.01E-10 |
| R1-2 | -2.183886389 | 0.008949437 | 0.035579468 |
| Stalker2 | -7.174233529 | 5.38E-08 | 1.10E-06 |

**A list of retrotransposons that are up regulated in the fat body in 5-day-old flies upon fat body-specific LAM depletion**

**Name of**

**retrotransposons fold change P-Value FDR**

ACCORD2 4.54936366 0.001020661 0.008318391

BLASTOPIA 6.031781017 1.99E-05 0.000464511

BURDOCK 4.202995505 0.000364365 0.003959431

DM1731 16.73707276 3.59E-10 2.92E-08

G6 18.30460731 1.79E-09 7.31E-08

GTWIN 5.10064423 0.001207933 0.009375862

Gypsy 4.275899175 0.000574518 0.005202579

Gypsy6 4.536826481 0.000814091 0.006984046

Gypsy7 4.409918189 0.007854129 0.04414562

IDEFIX 8.015517936 1.83E-06 5.97E-05

Invader4 5.776446296 8.98E-05 0.00133088

MICROPIA 3.376759117 0.004378183 0.029735158

QUASIMODO 3.842868818 0.001564034 0.011588073

ROVER 3.064187642 0.006800483 0.043895465

STALKER4 3.234957895 0.007540325 0.043895465

TAHRE 21.80904541 4.64E-11 7.57E-09

TIRANT 6.056026053 3.40E-05 0.000693705
