# Supplementary material for: Age‐associated de‐repression of retrotransposons in the Drosophila fat body, its potential cause and consequence
Source: Aging Cell. 2016 Apr 12;15(3):542–52. doi: 10.1111/acel.12465 (PMC4854910; doi:10.1111/acel.12465)
Supplement: Supplementary file 7 [file ACEL-15-542-s007.docx]

**Supporting Information**

**Supplemental Experimental Procedures**

***Chromatin immunoprecipitation (ChIP)-qPCR***

For ChIP experiments, adult fat bodies with attached dorsal cuticles of fly abdomen (some oenocytes, heart tube, and trachea were still attached) and larval fat bodies were dissected from 80, third instar female larvea, 5-day-old, 50-day-old female abdomens of *w^1118^* flies or 5-day-old *Lam^D395^/Lam^k2^* flies, respectively, in ice-cold Grace's Insect Media (Life Technologies #11605) and crosslinked with 1 ml cross-linking solution (2 % EM-grade formaldehyde, 50 mM Hepes pH 8.1, 1 mM EDTA, 0.5 mM EGTA, 100 mM NaCl) for 20 min at room temperature. The cross-linking was stopped by washing the tissues 3 times in ice-cold PBS buffer (Gibco #20012-027) with protease inhibitor (Thermo # PI-78425). The fat bodies were then resuspended in 1.2 ml SDS lysis buffer (0.1%SDS, 2mM EDTA, 20mM Tris-Cl pH 8.1) with protease inhibitor and incubated for 10 min on ice. To protect the ChIP DNA from loss, 25 μl pre-cross-linked yeasts (~ 1x 10^6^ cells) were added together with the SDS lysis buffer. The tissue lysate was then sonicated to shear DNA into 1000~2000 bp using a tip sonicator (Misonix sonicator 3000) followed by centrifugation for 10 min at 13,000 rpm and 4^o^C. The supernatants were diluted by adding 300 μl ChIP dilution buffer (5% Triton-100, 750mM NaCl) and divided into three 500 μl aliquots. One of the aliquots was set aside as the input sample and the other two was incubated with antibodies (8 μl rabbit polyclonal H3K9me3 antibody (abcam #ab8898) or 4 μl rabbit polyclonal H3K4me3 antibody (abcam #ab8580) or 8 μl control IgG (Santa Cruz #sc-2027)) at 4^o^C overnight. The mixtures were then incubated with 80 μl Protein G Dynabeads (Invitrogen, Cat. #100.03D) at 4^o^C for 4 hr. The protein G beads/antibody/histone/DNA complex was then washed once with 1 ml each of Low salt immune complex wash buffer (Millipore #20-154), High salt immune complex wash buffer (Millipore #20-155), LiCl immune complex wash buffer (Millipore #20-156), and then twice with 1 ml each of TE buffer (Millipore #20-157). The beads were then treated with 750 μl elution buffer (TE buffer with 0.25% SDS and 0.1M NaHCO_3_) at 65^o^C overnight. 30μl 5M NaCl was then added to the eluates and the set-aside input samples. The histone-DNA crosslink was reversed by heating at 65^o^C for 4 hr. 15μl of 0.5 M EDTA, 30μl 1M Tris-Cl pH 6.5, and 1.5μl of 20 mg/ml proteinase K (Life technologies #Am2546) were then added to each de-crosslinked samples followed by incubation at 45^o^C for 1 hr. To precipitate the ChIP-ed DNA, 30μg yeast tRNA (Life technologies #Am7119) was added as a carrier to each sample followed by phenol:chloroform:isoamyl alcohol (24:24:1) extraction and ethanol precipitation. The DNA pellet was re-suspended in 30 μl of TE buffer. Samples were analyzed using real-time PCR with iQ SYBR Green Supermix (Bio-rad #170-8880). ΔCt was used for assessing the relative level of each amplification product versus the amplification product of 5% of input DNA. The primers sets used for qPCR are listed below.

*Accord* (for H3K4me3 bingding):

L: ACAATCCACCAACAGCAACA

R: TTCAATCGAGTGTCGCAAAG

*Accord* (for H9K4me3 bingding):

L: GCAACCCCATAAACCAGCTA

R: CGGAAAATTCTGTTCCCTGA

*BATUMI* (for H3K4me3 bingding):

L: CATCCTGTCCGTGTATGTCG

R: GCACTGCCAAGAAGGATAGC

*BATUMI* (for H9K4me3 bingding):

L: GCCATTATGGTGCAACTCCT

R: CCTCTACTTGCCAGGTCTGC

*Copia2* (for H3K4me3 bingding):

L: GACAACGGGCGTGAGTATTT

R: CCTCAGCAACACAGACTGGA

*Copia2* (for H9K4me3 bingding):

L: CATTCTGCAAAAAGCGAACA

R: TCTCTCACCGCACAAATGAG

*Blastopia* (for H3K4me3 bingding):

L: CTGGTGTTAGTGCCTTGTCG

R: TGCTTGTATTTGGTCGGTGT

*Blastopia* (for H9K4me3 bingding):

L: GAGTCCCTTTCAAGCTCGTC

R: GCCAACTTCTCTTGGGACAT

*Gypsy6* (for H3K4me3 bingding):

L: CACTACCAGGCGGTCGTTAT

R: AGTCAGGCTTCCCTGTCTGA

*Gypsy6* (for H9K4me3 bingding):

L: GCGGTAGCCGCATATTACAT

R: GTCAGGCTTCCCTGTCTGAG

*Invider4* (for H3K4me3 bingding):

L: GTTTGACTGCTGACGTCGAT

R: CGAAACAGGAGTCTCATCCA

*Invider4* (for H9K4me3 bingding):

L: CTCCCAGTCGCATCATAGTG

R: GTTGAACAAAGCTCACGGAA

*QUSIMODO* (for H3K4me3 bingding):

L: AACCGCGAAGTTGTAATTGG

R: TGTGCCATGTTGTTGGCTAT

*QUSIMODO* (for H9K4me3 bingding):

L: ACTGGCTGGAAGCAAAAAGA

R: GCTGGTGTTCTCCCAGTTGT

*rp49* (for H3K4me3 and H3K9me3 bingding):

L: ATCGGTTACGGATCGAACAAGC

R: GTAAACGCGGTTCTGCATGAGC

***Chromatin immunoprecipitation (ChIP)-sequence***

For ChIP experiments, larval fat bodies were dissected from 200 third instar *w^1118^* or *Lam^D395^/Lam^k2^* larva in ice-cold Grace's Insect Media (Life Technologies #11605) and cross-linked with 1 ml cross-linking solution (2 % EM-grade formaldehyde, 50 mM Hepes pH 8.1, 1 mM EDTA, 0.5 mM EGTA, 100 mM NaCl) for 20 min at room temperature. The cross-linking was stopped by washing the tissues 3 times in ice-cold PBS buffer (Gibco #20012-027) with protease inhibitor (Thermo # PI-78425). The fat bodies were then resuspended in 1.2 ml SDS lysis buffer (0.1%SDS, 2mM EDTA, 20mM Tris-Cl pH 8.1) with protease inhibitor and incubated for 10 min on ice. To protect the ChIP DNA from loss, 20 μl pre-cross-linked yeasts (~ 1x 10^6^ cells) were added together with the SDS lysis buffer. The tissue lysate was then sonicated to shear DNA into 200~500 bp using a tip sonicator (Misonix sonicator 3000) followed by centrifugation for 10 min at 13,000 rpm and 4^o^C. The supernatants were diluted by adding 300 μl ChIP dilution buffer (5% Triton-100, 750mM NaCl) and incubated with 8 μl rabbit polyclonal H3K9me3 antibody (abcam #ab8898) or 4 μl rabbit polyclonal H3K4me3 antibody (abcam #ab8580) at 4^o^C overnight. The mixtures were then incubated with 80 μl Protein G Dynabeads (Invitrogen, Cat. #100.03D) at 4^o^C for 4 hr. The protein G beads/antibody/histone/DNA complex was then washed once with 1 ml each of Low salt immune complex wash buffer (Millipore #20-154), High salt immune complex wash buffer (Millipore #20-155), LiCl immune complex wash buffer (Millipore #20-156), and then twice with 1 ml each of TE buffer (Millipore #20-157). The beads were then treated with 70μl elution buffer (TE buffer with 0.25% SDS and 0.1M NaHCO_3_) at 65^o^C overnight. 30μl 5M NaCl was then added to the eluates. The histone-DNA crosslink was reversed by heating at 65^o^C for 4 hr. 2.5μl of 10% SDS, and 2.5μl of 20 mg/ml proteinase K (Life technologies #Am2546) were then added to each de-crosslinked samples followed by incubation at 45^o^C for 1 hr. The ChIP-ed DNA was extracted by phenol:chloroform:isoamyl alcohol (24:24:1) and precipitated by ethanol. The DNA pellet was re-suspended in 60 μl of TE buffer. Then, the fragmented DNA solutions were used for building the sequencing libraries. We used the reagents in Illumina TruSeq RNA sample prep kit V2 (Illumina # RS-122-2001) and followed its Low Sample protocol to achieve the steps of building DNA sequencing library including end repair, 3’ adenylation, adapter ligation, and enrich DNA fragments. The libraries were sequenced by single end 50-bp reads on Illumina HiSeq 2000.

***Bioinformatics***

RNA-seq and ChIP-seq fastq files were generated by Illumina Pipeline (Casava 1.8). ChIP-seq reads were mapped to the *Drosophila* genome dm3 using bowtie with parameter “-v 2 -m 1”. 3,794,277 of 126,524,246 H3K4me3 reads (3.00%), and 11,793,488 of 77,788,276 H3K9me3 reads (15.16%) were mapped uniquely to the dm3 genome for Larvae fat body. These percentages were expected because of yeast chromatin carrier protection. RNA-seq reads were mapped to the dm3 genome using Tophat2 ([Kim et al., 2013](#_ENREF_2)). Refseq annotation downloaded from the UCSC genome browser was supplied to Tophat2 for exon junction information.

Sequences of retrotransposons were downloaded from the UCSC genome browser by selecting repeat masker in the table browser. Sequences of the same retrotransposon on different positions of the genome were concatenated to form an artificial genome of retrotransposons. Then ChIP-seq and RNA-seq data were mapped to the artificial genome using bowtie with parameter “-v 2 -m 1”. If a read is also mapped to other non-repeat region of the genome, the read is discarded. A custom script was used to count the number of reads mapped to each retrotransposon. Then we used edgeR ([Robinson et al., 2010](#_ENREF_5)) to call differentially expressed retrotransposons (fold change≥2 and FDR<0.05). For ChIP-seq, we used the number of reads that mapped uniquely onto the genome as normalization factors for edgeR. For RNA-seq, we used the number of reads mapped to normal mRNAs as normalization factors for edgeR.

**Supplemental Figure and Table Legends**

**Figure S1 (related to Figure 2). The expression of *AGO2* significantly increased in fat bodies in the *Cg-gal4-*driven** ***AGO2* EP line compared to controls.**

qRT-PCR analyses of *AGO2* in the indicated young (5 day) fat bodies. The expression of *AGO2* is significantly increased in the *AGO2* overexpressing fat bodies (*Cg-gal4/+;tub-gal80^ts^/UAS-AGO2^EP^*) compared to the Gal4 (*Cg-gal4/+;tub-gal80^ts^/+*) and UAS (*+/+;tub-gal80^ts^/ UAS-AGO2^EP^*) controls. The fold expression change was plotted relative to 5-day-old control fat bodies (*Cg-gal4/+; tub-gal80^ts^/+*), which was set to 1. Error bars, SEM based on three independent experiments. Student’s t test: ^*p*>0.05, ** *p*<0.01.

**Figure S2 (related to Figure 3). The expression of retrotransposons does not significantly change in young fat bodies in the control flies with *Cg-gal4-*driven** ***GFP* RNAi as compared to flies carrying no *GFP* RNAi*.***

qRT-PCR analyses of the indicated retrotransposons in young (5 day) fat bodies depleted of GFP (*Cg-gal4/+;tub-gal80^ts^/GFP RNAi*). The expression of the tested retrotransposons in young fat bodies depleted of GFP (*Cg-gal4/+;tub-gal80^ts^/GFP RNAi*) does not change compared to the control (*Cg-gal4/+;tub-gal80^ts^/+*). The fold expression change was plotted relative to 5-day-old control fat bodies (*Cg-gal4/+; tub-gal80^ts^/+*), which was set to 1. Error bars, SEM based on three independent experiments. Student’s t test: ^*p*>0.05.

**Figure S3 (related to Figure 5). The expression of *Dicer-2* or *AGO2* did not significantly change either in aged fat bodies or lamin-B depleted young fat bodies.**

**A**. RNA-seq showed that the expression of *Dicer-2* and *AGO2* in old fat bodies or LAM depleted young fat bodies was similar to the wild type young fat bodies.

**B**. qRT-PCR analyses of the expression of *Dicer-2* or *AGO2* in wild-type young (5 day) fat bodies, wild-type old (50 day) fat bodies, young Gal4-control fat bodies, young UAS-control fat bodies, or young fat bodies depleted of LAM (*Cg-gal4/+; tub-gal80^ts^/Lam RNAi*). The fold expression change was plotted relative to 5-day-old wild-type fat bodies, which was set to 1. Error bars, SEM based on three independent experiments. Student’s t test: ^*p*>0.05.

**C**. A box-plot showing the distribution of the Log_2_-fold change for all 111 retrotransposons based on the ChIP-seq of the third instar larval fat bodies from the *Lam-/-* (*Lam^D395^/Lam^k2^*) and wild type animals. The large boxes in the graph show the 25% and 75% quantiles; the line in each box shows the median; the small square in the middle of each box shows the mean; the upper and lower whiskers show the 90% and 10% quantiles; and the stars show the maximum and minimum values. Thus the plot shows that >75% of all analyzed retrotransposons exhibit increased H3K4me3 and decreased H3K9me3 in lamin-null fat bodies.

**Figure S4 (related to Figure 5). ChIP-qPCR analyses of H3K4me3 and H3K9me3 on selected retrotransposons in fat bodies.**

**A.** ChIP-qPCR analysis of selected retrotransposons (*Accord, BATUMI, Copia2 and Gypsy6*) that become de-repressed upon LAM depletion. Fat bodies were dissected from the third instar larvae of wild type (*w^1118^*) or *Lam-/-* (*Lam^D395^/Lam^k2^*) flies. Chromatin was immunoprecipitated with antibodies to H3K4me3, H3K9me3, or control IgG. Primers corresponding to *Accord, BATUMI, Copia2, Gypsy6*, and rp49 (control) were used to amplify the precipitated DNA. ChIP samples were normalized to the input DNA. Error bars, SEM, based on three independent experiments. Student’s t-tests: ^*p*>0.05, **p*<0.05, ***p*<0.01.

**B.** ChIP-qPCR analysis of *Gypsy6* and *QUASIMODO* retrotransposons that become de-repressed upon aging. Fat bodies were dissected from 5-day or 50-day wild type (*w^1118^*), or 5-day *Lam-/-* (*Lam^D395^/Lam^k2^*) flies. Chromatin was immunoprecipitated with antibodies to H3K4me3, H3K9me3, or control IgG. Primers corresponding to *Gypsy6*, *QUASIMODO*, and rp49 (control) were used to amplify the precipitated DNA. ChIP samples were normalized to the input DNA. Error bars, SEM, based on three independent experiments. Student’s t-tests: ^*p*>0.05, **p*<0.05, ***p*<0.01.

**Table S1 (related to Figure 1). A list of retrotransposons that were down regulated in wild type 50-day-old fat bodies as compared to that of the young (5-day).**

Retrotransposons, fold changes, p values (by hypergeometric test), and FDR (False Discover Rate) are indicated.

**Table S2 (related to Figure 3). A list of retrotransposons that were up regulated in 5-day old fat bodies upon LAM depletion.**

Retrotransposon, fold change, P values (by hypergeometric test), and FDR (False Discover Rate) are indicated.
